# Post-COVID-19 Parkinsonism and Parkinson’s Disease Pathogenesis: The Exosomal Cargo Hypothesis

**DOI:** 10.3390/ijms23179739

**Published:** 2022-08-28

**Authors:** Dimitrios S. Mysiris, George D. Vavougios, Eirini Karamichali, Stamatia Papoutsopoulou, Vasileios T. Stavrou, Eirini Papayianni, Stylianos Boutlas, Theodoros Mavridis, Pelagia Foka, Sotirios G. Zarogiannis, Konstantinos Gourgoulianis, Georgia Xiromerisiou

**Affiliations:** 1 Faculty of Medicine, University of Thessaly, 41110 Larissa, Greece; 2 Department of Neurology, Faculty of Medicine, University of Cyprus, Lefkosia 1678, Cyprus; 3 Laboratory of Pulmonary Testing and Rehabilitation, Department of Respiratory Medicine, Faculty of Medicine, University of Thessaly, 41110 Larissa, Greece; 4 Molecular Virology Laboratory, Hellenic Pasteur Institute, 11521 Athens, Greece; 5 Department of Biochemistry and Biotechnology, Faculty of Life Sciences, University of Thessaly, Mezourlo, 41500 Larissa, Greece; 6 1st Neurology Department, Eginition Hospital, Medical School, National & Kapodistrian University of Athens, 11528 Athens, Greece; 7 Department of Physiology, Faculty of Medicine, University of Thessaly, Biopolis, 41500 Larissa, Greece; 8 Department of Neurology, University Hospital of Larissa, Faculty of Medicine, School of Health Sciences, University of Thessaly, 41110 Larissa, Greece

**Keywords:** Parkinson’s disease, SARS-CoV-2, exosomes, neuroinflammation, inflammation, parkinsonism, alpha-synuclein, post-COVID-19, neurodegeneration, virus

## Abstract

Parkinson’s disease (PD) is the second most prevalent neurodegenerative disease after Alzheimer’s disease, globally. Dopaminergic neuron degeneration in substantia nigra pars compacta and aggregation of misfolded alpha-synuclein are the PD hallmarks, accompanied by motor and non-motor symptoms. Several viruses have been linked to the appearance of a post-infection parkinsonian phenotype. Coronavirus disease 2019 (COVID-19), caused by emerging severe acute respiratory syndrome coronavirus-2 (SARS-CoV-2) infection, has evolved from a novel pneumonia to a multifaceted syndrome with multiple clinical manifestations, among which neurological sequalae appear insidious and potentially long-lasting. Exosomes are extracellular nanovesicles bearing a complex cargo of active biomolecules and playing crucial roles in intercellular communication under pathophysiological conditions. Exosomes constitute a reliable route for misfolded protein transmission, contributing to PD pathogenesis and diagnosis. Herein, we summarize recent evidence suggesting that SARS-CoV-2 infection shares numerous clinical manifestations and inflammatory and molecular pathways with PD. We carry on hypothesizing that these similarities may be reflected in exosomal cargo modulated by the virus in correlation with disease severity. Travelling from the periphery to the brain, SARS-CoV-2-related exosomal cargo contains SARS-CoV-2 RNA, viral proteins, inflammatory mediators, and modified host proteins that could operate as promoters of neurodegenerative and neuroinflammatory cascades, potentially leading to a future parkinsonism and PD development.

## 1. Introduction

Parkinsonism is a clinical syndrome defined by the presence of resting tremor, bradykinesia, rigidity and postural instability [1]. These motor symptoms are characteristically observed in Parkinson’s disease (PD) [2], which remains the primary cause of parkinsonism, but there are other disorders with the same symptoms that mirror it [3,4]. PD is the second most prevalent neurodegenerative disease worldwide after Alzheimer’s disease (AD) and constitutes a debilitating, progressive motor disorder characterized by degeneration of the nigrostriatal dopaminergic pathway [5]. The prevalence of PD is estimated to be approximately 0.5–1% among those 65–69 years of age, rising to 3% among persons of 80 years and older [6], with an annual incidence rate of approximately 11–19/100,000 cases per year [7,8]. Although PD is generally an idiopathic disorder, there is 5–10% of PD cases that report a family history or display a clear Mendelian inheritance [9,10]. The incremental loss of dopaminergic neurons in the substantia nigra pars compacta (SNpc) and striatum is the mechanistic cause of motor manifestations, with 60–70% dopaminergic neuron loss required for the appearance of motor symptoms [11]. However, prior to motor manifestation onset, patients may display non-motor symptoms such as hyposmia, gastrointestinal dysfunction, and sleep disorders [12]. The neuropathological hallmark of PD is the misfolding and aggregation of alpha-synuclein (α-syn), which is the major protein component of Lewy bodies (LB). Indeed, formation of α-syn protein clumps within neural cells triggers the initiation of neurodegeneration processes [5].

PD is a disease of multicomplex etiology, involving the interaction of aging, genetics, and environmental variables, as well as infectious agents, such as viral infections [13,14]. Additionally, there is now a wide range of data to support the existence of viral parkinsonism, which often manifests following recovery from viral infections [4]. Although the precise mechanisms remain unclear, viruses have been implicated as potential etiological or trigger factors for both PD pathogenesis [15,16] and viral parkinsonism [4]. Recent data suggest that the emerging human severe acute respiratory syndrome coronavirus 2 (SARS-CoV-2), responsible for the ongoing pandemic that has already killed more than 6.4 M people worldwide [17], may be one of these viruses [18,19,20].

On cellular and molecular level, mitochondrial dysfunction, defective autophagy, oxidative stress, and neuroinflammation are all thought to play a role in PD pathogenesis and they are linked to the accumulation and spread of misfolded α-syn [21,22,23]. The “prion-like” cell-to-cell dissemination of amyloidogenic proteins, such as α-syn, principally refers to the formation and subsequent spread of self-propagating pathological α-syn aggregates throughout brain regions and has lately garnered considerable attention in the quest to understand PD pathophysiology [24,25,26,27]. Several in vitro studies, both in animals and continuous human cell lines, have supported this reminiscent of, yet distinct from prion diseases, mechanism of misfolded α-syn spread [28,29,30]. Exosomes, the nanosized vesicles and masters of intercellular communication [31], have been proposed to serve as an efficient “vehicle” of transportation for such proteins [32], mainly because they are a priori involved in several homeostatic procedures in the central nervous system (CNS) including myelination maintenance, synaptic plasticity, antigen presentation, signal transduction, neurogenesis, and trophic support for neurons [33,34]. Interestingly, many viruses, including SARS-CoV-2, have been shown to regulate exosomal biogenesis and cargo content upon release from infected host cells [35,36]. On top of that, findings from our group and others suggest that a virus-manipulated exosomal cargo could become a blueprint for disease progression even after the complete eradication of the viral agent, due to either immune response-related or drug-mediated viral clearance [37,38]. Since exosomes have a prominent position in pathogenesis and diagnosis of neurodegenerative diseases [32] and are known to be closely linked both to viral infection establishment and infectious disease progression even after virus eradication [39,40], we aim in this review to point out and discuss potential exosome-mediated mechanisms that could lead to post-COVID-19 parkinsonism and PD pathogenesis.

## 2. Viral Ιnfections as Τriggers for Parkinsonism and PD Development

Several studies have demonstrated that viruses may contribute to the etiology of PD and parkinsonism, despite the fact that the underlying molecular and cellular mechanisms remain obscure. The first recorded association between viral infections and parkinsonism was observed during the Spanish flu and the appearance of encephalitis lethargica, an unknown disease with parkinsonian phenotype in survivors [41]. Major human viruses, such as hepatitis C virus (HCV) [42], herpes simplex virus-1 (HSV-1) [43], human immunodeficiency virus (HIV) [44], varicella-zoster virus (VZV) [45], West Nile virus (WNV) [46], Japanese encephalitis virus (JEV) [47,48], and Epstein–Barr virus (EBV) [49], have all been cited as risk factors for PD development or parkinsonism [3]. Notably, the role of influenza A virus (IAV) in the etiology of the transient parkinsonian phenotype [50] and in PD development [3] has been documented in several in vivo and especially in vitro studies. A case-control study found that an influenza diagnosis was linked to PD development 10 years following infection onset [51], while IAV was found postmortem in the substantia nigra of PD patients [52]. Furthermore, H5N1 infection in a mouse model resulted in Parkinson’s phenomenology, sustained microglial activation, and α-syn aggregation, leading to dopaminergic neuron loss in SNpc [53]. Similarly, H1N1 infection in mice resulted in persistent microglial activation as a sign of chronic virus-induced neuroinflammation that could potentially lead to neurodegeneration [54]. More recently, another in vitro study has demonstrated that H1N1 replication can directly disrupt protein homeostasis, inducing α-syn aggregates in Lund human mesencephalic dopaminergic cells, but failing to regulate TAR DNA-binding protein 43 (TDP-43) or tau protein. Those results clearly hint at a selective effect of H1N1 virus on α-syn misfolding [55].

The key pathophysiological processes by which viruses contribute to parkinsonism development remain unclear; however, direct neuronal damage, sustained neuroinflammation, cerebral edema due to virus-mediated damage of brain endothelium, and induction of α-syn aggregation have all been proposed as crucial neurobiological pathways of dopaminergic neuron loss and α-syn pathology [3]. Notably, due to its tendency to entrap viral particles and reduce viral replication, α-syn has been postulated to be a natural antiviral defense mechanism for neurons [56]. This notion was supported by in vivo experiments, where WNV-infected α-syn-knockout mice showed decreased survival compared to the control group [57]. Additionally, it has been suggested that viruses can cause α-syn aggregation and oligomerization through molecular mimicry mechanisms [58,59]. Taken together, these observations strongly support the notion that virus-mediated neuronal deposition of pathological α-syn may induce neurotoxicity and PD pathology.

The relationship between other members of the human *Coronaviridae* family, such as OC43 and 229E, and PD has been previously described, since antibodies against these coronaviruses were found in the cerebrospinal fluid (CSF) of PD patients [60]. The novel coronavirus SARS-CoV-2 emerged in China at the end of 2019 and triggered an outbreak of atypical viral pneumonia [61]. Due to its enhanced transmissibility, this unusual coronavirus disease, also known as coronavirus disease 2019 (COVID-19), marched fast over the world, constituting a huge public health burden [62,63]. SARS-CoV-2 spreads via infected secretions, such as saliva and respiratory droplets, through direct, indirect, or close contact with infected patients, even if COVID-19 symptomatology is absent [64,65]. While symptoms of COVID-19 are primarily systemic or respiratory, several studies demonstrate the presence of a broad spectrum of neuropsychiatric consequences including anosmia, ageusia, altered consciousness, headache, seizures, and paresthesias [66,67,68]. Several studies have shown that COVID-19-related neurological sequelae might persist long after the acute phase of infection [69]. The term “long” or “post”-COVID-19 syndrome refers to a syndrome observed after the acute infection period and it is characterized by the presence of a combination of COVID-19-related symptoms lasting for more than 12 weeks [70]. These symptoms cannot be explained by an alternative diagnosis and are considered a disability under the Americans with Disabilities Act (ADA) [71]. The post-COVID-19 syndrome includes a plethora of neurological manifestations such as fatigue, brain fog, cognitive impairment, and olfactory dysfunctions [72,73,74], many of which are also present in PD [2]. Thus, since SARS-CoV-2 shares immunopathological similarities with other viruses linked to parkinsonism, such as influenza [75], and because of COVID-19-related neurological consequences, it is reasonable to suspect that these persistent symptoms might be a prologue to a post-COVID-19 new-onset neurological disease.

## 3. SARS-CoV-2 Infection and PD Overlaps

### 3.1. Clinical Co-Manifestations

To date, only few cases of parkinsonism have been reported in literature following COVID-19 infection [19,20,76,77,78]. In these studies, the authors speculate a possible causative link between COVID-19 infection and a post-COVID new-onset parkinsonian phenotype, but they do not address the possibility of prodromal, pre-symptomatic PD, which became symptomatic as a result of biological or psychological stress processes associated with COVID-19. In the latter case, SARS-CoV-2 infection could act as a trigger that unmasks an underlying PD phenotype, possibly by stimulating neuroinflammatory and neurodegenerative cascades. In addition, SARS-CoV-2 infection has been demonstrated to significantly worsen motor and non-motor symptoms in people with pre-existing PD [79,80]. Considering the prevalence of post-COVID-19 syndrome [81,82], a multicenter study found that 23 out of 27 PD patients developed post-COVID-19 symptoms, with the most common long term effects of COVID-19 being the deterioration of motor function and the requirement for increased levodopa daily dose, followed by fatigue, cognitive disturbances including brain fog, and sleep disorders [83].

Probably the clinical symptoms most commonly shared between PD and COVID-19 are gustatory and especially olfactory dysfunctions. Indeed, both olfactory and gustatory impairments are among the earliest non-motor PD features [84,85]. Surprisingly, these are common early onset symptoms of COVID-19 and it has been observed that hyposmia–anosmia and dysgeusia could persist long after viral load decline, constituting a key clinical manifestation of the long COVID-19 syndrome [86,87]. Due to lack of evidence regarding the definite CNS infiltration, the olfactory route is discussed as a way for SARS-CoV-2 to gain access to the CNS. Indeed, a postmortem study demonstrated that the highest levels of SARS-CoV-2 RNA and spike protein (S protein) among various brain areas were found in the olfactory mucosal–nervous milieu, as well as in neuroanatomical areas related to the olfactory tract. In this regard, the olfactory mucosa could serve as an “anatomical bridge” for SARS-CoV-2 CNS invasion through axonal transport [88]. Furthermore, angiotensin-converting enzyme 2 (ACE2), an essential cell surface receptor responsible for S protein-mediated entry of SARS-CoV-2, was found to be expressed by epithelial cells of the human olfactory mucosa [89]. The extent of α-syn pathology in other brain regions has been substantially linked with the pathological burden in the olfactory bulb, suggesting that PD pathology extends along olfactory pathways [90]. The Braak hypothesis proposes that LB are initially found in olfactory structures, such as the olfactory bulb, and then they gradually spread towards the brain stem and ultimately to the cerebral cortex, strengthening the scenario that the earliest lesions could develop at non nigral areas [91,92]. Accordingly, Beach and colleagues have demonstrated that the olfactory bulb constitutes a primary affected area in α-synucleinopathies, including PD. In fact, it was suggested that the extent of α-synucleinopathy in the olfactory bulb strongly predicts the neuropathological confirmation of PD and reflects the severity of α-synucleinopathy in other brain regions [93]. Based on these studies, one could hypothesize that the olfactory route might pose a way for SARS-CoV-2 to gain access to the CNS, where it can modify neuropathological pathways pertinent to PD development.

Another common pathology shared between PD and COVID-19 is the deregulation and dysfunction of the gastrointestinal (GI) tract. GI symptoms and intestinal inflammation may emerge years before clinical indications of PD become apparent [94,95]. Specifically, gastrointestinal dysbiosis has been proposed to be involved in PD pathogenesis [96] and the enteric nervous system has been previously identified as a primary region for abnormal α-syn aggregation, which may then spread from the periphery to the CNS [97,98,99]. Specifically, the dorsal motor nucleus of the vagus nerve (DMV) receives signals from vagal parasympathetic neurons that project to the entire GI system. The DMV is involved in the PD–neuroanatomical pathway, since a monosynaptic nigro–vagal pathway that connects the SNpc to the DMV has been identified in the rat [100]. In postmortem PD studies, the DMV and the vagus nerve itself are among the most frequently afflicted structures [101,102] and they constitute principal areas of LB accumulation, even at the earliest stages of disease development [91]. In vitro research has shown that pathological α-syn may spread from the gut to the brain through the vagus nerve, with DMV being the first area of the brain to be impacted. From there, α-syn can spread to other PD brain regions including the SNpc, resulting in dopaminergic neuron loss and the appearance of the parkinsonian phenotype [103]. Interestingly, the vagus nerve has been proposed as a pathway through which SARS-CoV-2 can retrogradely invade the CNS, thus enhancing its neuroinvasiveness [104,105].

Importantly, other GI manifestations, such as diarrhea, emerged as common clinical symptoms of COVID-19, while SARS-CoV-2 RNA detection in fecal samples may persist post-infection [106]. On top of that, gut microbiota imbalance due to extrapulmonary SARS-CoV-2 infection has also been observed in COVID-19 [107,108]. This warrants further investigation because GI microbiota equilibrium plays an important role in several physiological processes ensuring brain integrity and neurogenesis [109,110]. Taken together, the above observations suggest that SARS-CoV-2 infection could promote PD development and progression through a virus-exerted dysfunction of the GI system.

### 3.2. Inflammatory and Molecular Overlapping Pathways

Common inflammatory events unraveling during PD development and observed in the acute phase of SARS-CoV-2 infection, as well as after COVID-19 remission, may indicate a link between these two disorders. Virus-mediated sustained or aberrant neuroinflammation could be a decisive pathobiological process for the initiation of a neurodegenerative disease, such as PD, long after recovery from the viral infection [111,112,113]. Indeed, growing evidence indicates that SARS-CoV-2 induces neuroinflammation [114] through its neurotropic, neuroinvasive, and neurovirulence effects [115,116] or even via immune-mediated pathways [117]. SARS-CoV-2 infection also triggers systemic inflammatory responses and induces cytokine release [118]. Severe COVID-19 is characterized by a cytokine storm syndrome, which is a major cause of mortality [118,119]. Several studies have demonstrated the presence of inflammatory mediators, such as increased levels of pro- and anti-inflammatory interleukins (IL-1, IL-2, IL-6, IL-10) and tumor necrosis factor-alpha (TNF-α) in the serum of COVID-19 patients [120,121,122,123]. Interestingly, a small prospective observational study had previously found that high levels of IL-6 were linked to a higher chance of developing PD [124]. Evidently, an exacerbated systemic infection that causes a huge release of inflammatory mediators, including cytokines, chemokines, and antibodies, could lead to increased blood–brain barrier (BBB) permeability [125]. Functional and structural integrity of the BBB is pivotal in maintaining brain homeostasis [126]. A neurovascular unit (NVU) consists of multiple cell types, including brain microvascular endothelial cells (BMVECs), astrocytes, pericytes, microglia, and neurons, connected together with extracellular matrix components, and is a rigorous regulator of BBB permeability [127]. NVU disruption has been previously associated with neurodegenerative diseases [128]. In particular, BMVECs constitute an important component of NVU and are intricately interconnected through tight junction (TJ) proteins. However, inflammation affects BBB integrity and stability mainly through cytokine-induced degradation of TJ proteins [129]. SARS-CoV-2-mediated brain endothelial inflammation, upregulation of inflammatory mediators, and most significantly, disruption of BBB stability, have also been observed in human BMVECs [130]. According to in vitro studies, SARS-CoV-2 was shown to infect human BMVECs and cause a decrease in TJ protein expression [130,131]. Furthermore, incubation of human BMVECs with S protein resulted in enhanced ACE2 expression, thereby facilitating viral entry and inducing neuroinflammation [132].

When BBB becomes impaired, pro-inflammatory cytokines and factors, innate immune cells from the periphery, and SARS-CoV-2 could possibly pass through and infiltrate the CNS. In that case, the CNS professional immune cells, microglia and astrocytes, may also become activated [133,134]. Neuroinflammation is then likely to set in fast, leading to elevated production of cytokines, chemokines, reactive oxygen species (ROS), and secondary messengers [135]. Microglia, which are highly susceptible to pro-inflammatory stimuli, are concentrated in areas harboring dopaminergic neurons, making them particularly vulnerable to inflammatory mediators [136,137]. Interestingly, the S1 subunit of S protein was found to efficiently trigger neuroinflammation, including microglia activation, release of multiple pro-inflammatory cytokines, and cause behavioral deficits in rats [138]. Consequently, these neuroinflammatory cascades lead to enhanced apoptotic activity, increased ROS levels, mitochondrial dysfunction, and eventually neurodegeneration [139,140].

Finally, cellular senescence is a core homeostatic event that provides yet another, age- and state-dependent substrate for neurodegeneration and the development of diseases like AD and PD [141,142]. Cellular senescence in the aging brain affects both neuronal and non-neuronal cells, and it is characterized by a broad array of interconnected disruptions, such as disruptions in autophagy, bioenergetics, and mitochondrial dynamics, as well as the onset of low-grade inflammation [142]. This cumulative array of dysfunction culminates in the accumulation of proteopathic seeds, including tau, amyloids, and α-syn, and tissue-wide remodeling [141]. It has been shown that SARS-CoV-2 infection induces “immunosenescence” and enhances the senescence-associated secretory phenotype (SASP) in infected tissues, via disruption of host antiviral mechanisms, such as interferon signaling pathways [143,144,145]. Taken together, all the aforementioned studies strongly indicate that the COVID-19 cytokine storm and innate immunity dysregulation may cause neuroinflammation and, in consequence, neurodegeneration.

Neuropathological findings in postmortem brain tissues from COVID-19 patients further support the involvement of COVID-19-related neuroinflammatory processes in PD development. A postmortem brain study of 43 COVID-19 patients has shown activation of microglia and CNS infiltration by cytotoxic T-lymphocytes, more apparent in the brainstem [146]. Regardless of COVID-19 disease severity, significant inflammatory responses such as astrogliosis, microglia activation, and perivascular T-lymphocyte infiltration were observed postmortem in both white and gray matter of patient brains [147]. Performing single-nucleus RNA sequencing and immunohistochemistry on tissue from a group of individuals who died with COVID-19 and a group of individuals who died from other causes, Yang and colleagues revealed glia transcriptomic changes that indicated a COVID-19-associated activation of inflammatory pathways. The ensuing dysregulation of homeostatic pathways could potentially lead to neurodegeneration [148]. Specifically, microglia and astrocytic subpopulations were enriched by inflammatory genes and deregulated neuroprotective ones that had been previously linked to PD and other human neurodegenerative diseases, such as the glial fibrillary acidic protein (GFAP), the interferon-induced transmembrane protein-3 (IFITM3), and others [149,150].

Another mechanism that may contribute to PD pathogenesis involves the renin–angiotensin system and ACE2, which are implicated in the pathophysiology of COVID-19 and may play a role in neuroinflammation-mediated neurodegeneration in PD [151,152]. ACE2 is highly expressed in several brain areas [153], including striatum [154], the substantia nigra, the olfactory bulb [155], and the brain endothelium [130,156,157]. Induced pluripotent stem cells (IPCS) derived from midbrain dopaminergic neurons were shown to be vulnerable to SARS-CoV-2 infection in vitro [158], unravelling the potentially direct neurotrophic effect of SARS-CoV-2 in strategic PD areas. Furthermore, SARS-CoV-2-induced Toll-like receptor (TLR) overactivation led to ACE2 upregulation and promoted the neurotrophic and neuroinflammatory outcomes of SARS-CoV-2 infection [159]. TLRs belong to the family of innate immune receptors and play an important role in the activation of innate immunity, including activation of glial cells. TLR-mediated stimulation of intracellular signaling pathways culminates in the release of proinflammatory mediators such as IL-6, IL-1, TNF-a, and nuclear factor-κB (NF-κB) [160]. Protein-to-protein interaction between SARS-CoV-2 S protein and TLR-4 has been previously recorded [161]. SARS-CoV-2-mediated overactivation of the TLRs may lead to hyperinflammation, ACE2 upregulation and microglia switching from the neuroprotective to the neurotoxic phenotype [159,162]. In sequel, sustained gliosis and prolonged neuroinflammation could lead to α-syn aggregation and finally loss of dopaminergic neurons in the SNpc [112].

Aside from neuroinflammation, dysregulation of several homeostatic molecular pathways has been identified in PD onset and development. These alterations also occur during host–virus interactions as the virus attempts to direct critical cellular infrastructure towards completion of its own lifecycle. SARS-CoV-2 viral proteins were shown to post-translationally reconfigure the biological function of 24 host proteins expressed in lung. The latter act as perturbators and interact with 44 CNS proteins that are known to be implicated in PD pathogenesis [163]. Specifically, SARS-CoV-2-mediated deregulation of Rab7a and nucleoporin-62 (NUP62) could be strongly involved in PD pathogenesis, because Rab7 lysosomal protein decreases α-syn aggregation and associated neurotoxicity [164], while NUP62 is crucial for autophagosome development [165]. Furthermore, SARS-CoV-2 proteins can interact and bind to a variety of human protein trafficking molecules. Protein trafficking, translation, transcription, and ubiquitination regulation are all coordinated by these biomolecules, leading to neuroprotection, protection of BBB integrity, and neurogenesis [166]. A recent study demonstrated a direct interaction between SARS-CoV-2 nucleocapsid protein (N-protein) and α-syn, which led to the aggregation of the latter into amyloid fibrils, a highly pathogenic form of the protein, linked to PD. Co-administration of SARS-CoV-2 N protein and α-syn to a PD cell model resulted in twice the neuron loss due to neurotoxicity compared to control cells treated with α-syn alone [167].

Other important cellular processes implicated in the loss of dopaminergic neurons in SNpc are thought to be oxidative stress and mitochondrial dysfunction, endoplasmic reticulum stress, and the impairment of protein degradation systems [168,169,170].

A key molecular factor in PD development and progression is mitochondrial dysfunction and oxidative stress [171,172]. An imbalance between ROS generation and cellular antioxidant activity leads to oxidative stress and ROS can further affect mitochondria, attenuating adenosine triphosphate (ATP) production as well as causing damage to mitochondrial DNA [173]. In addition to causing direct cellular damage, oxidative stress can speed up neuron degeneration by inducing inflammatory or apoptotic pathways, such as NF-κB or caspase activation [174]. In PD studies, mitochondrial dysfunction may occur months before the onset of striatal dopaminergic neuron loss [175] and PD patients have been well documented to possess reduced or deficient mitochondrial complex I activity in the SNpc [176,177]. In mice, accumulation of wild-type α-syn in dopaminergic neurons reduced mitochondrial complex I activity and elevated ROS production, leading to cell death [178]. SARS-CoV-2 seems to interact with and manipulate mitochondria in order to hijack and evade mitochondria-mediated immune response for its own replication and survival [179,180]. In this effort, SARS-CoV-2 may induce mitochondrial impairment [181,182], mitochondria-mediated oxidative stress, and mitochondrial damage through mitochondrial membrane depolarization, mitochondrial permeability transition pore opening, and enhanced ROS release [183,184,185]. Furthermore, the virus prevents mitophagy by blocking the binding of p62 and microtubule-associated protein 1A/1B-light chain 3 (LC3), thereby hindering viral RNA breakdown [185].

Finally, mitochondria aid the antiviral immune response by allowing release of pro-inflammatory cytokines [186]. ACE2 has been suggested to regulate mitochondrial function [187]. Its expression is decreased when SARS-CoV-2 S protein binds to ACE2 on microglia cells, causing ATP reduction and activation of the ROS-generating enzyme NADPH oxidase [188]. The ensuing increase in ROS production and oxygen consumption may lead to neuroinflammation and loss of neighbor dopaminergic neurons [189].

Endoplasmic reticulum (ER) stress has been linked to neurodegenerative diseases, including PD [190,191]. ER homeostasis disruption and extended ER stress lead to misfolded protein accumulation and may stimulate particular proapoptotic pathways through the activation of the transcription factor C/EBP homologous protein (CHOP) and cysteine proteases caspase-4/12 [192,193]. Growing evidence suggests that SARS-CoV-2 proteins interact with the ER compartment and may induce ER stress [194,195]. SARS-CoV-2 open reading frame 8 (ORF8) is capable of inducing ER stress by triggering the activating transcription factor 6 (ATF6) and inositol-requiring enzymes 1 (IRE1) branches of the ER stress pathway [196], potentially leading to α-syn accumulation [197]. Aside from initiating apoptotic pathways, ER stress is a powerful stimulator of NF-κB activation and inflammatory gene transcription [198,199]. SARS-CoV-2 also appears to activate NF-κB, causing inflammation, possibly through ER stress or via interaction with the non-structural protein Nsp5 [200]. Notably, NF-κB is a crucial transcription factor that regulates inflammation and dopaminergic neurons loss in PD patients [201]. Hence, deregulation of this signaling pathway has been linked to PD onset and pathology [202] by favoring α-syn accumulation, aggregation, and spreading, oxidative stress-induced neuron apoptosis, neuroinflammation, and dopaminergic neuron loss [139,203,204].

When aggregation and deposition of misfolded α-syn elicit dopaminergic neuron loss, protein degradation systems come to the rescue. The ubiquitin–proteasome system (UPS) and the autophagy–lysosomal pathway (ALP) are important proteolytic systems in neurons and critical for refolding or elimination of misfolded proteins; therefore, they play a significant role in cellular homeostasis [205]. Impairment or even failure of these systems may contribute to PD pathogenesis and progression [21,206]. SARS-CoV-2 virulent components, such as ORF proteins, seem to modify autophagy formation and function, leading to SARS-CoV-2-induced autophagy disruption and potentially neuron damage [207,208]. Specifically, ORF3a was shown to impede autophagosome–lysosome (A-L) fusion and ALP formation by interacting directly with the VPS39 subunit of the homotypic fusion and protein sorting (HOPS) complex. ORF3a further damages lysosomes and impairs their function. Remarkably, this feature of HOPS-VPS39-mediated A-L fusion inhibition appears to be unique to SARS-CoV-2, since the quite similar ORF3a of SARS-CoV was ineffective in inhibiting A-L fusion [209]. Furthermore, another study found that although ORF7a protein stimulates autophagy, it also limits A-L fusion progression by downregulating the SNAP29 protein via caspase 3 (CASP3) activation, providing a mechanism through which SARS-CoV-2 uses the autophagic system to facilitate its own propagation [210]. Interestingly, a SARS-CoV-2 papain-like protease has been identified to directly cleave serine/threonine unc-51-like kinase (ULK1) and prevent ULK1-ATG13 complex formation [211]. ULK1 is an upstream autophagy orchestrator, which phosphorylates key regulatory proteins in autophagosome formation [212]. In this regard, ULK1 cleavage is expected to completely inhibit the ALP function, due to lack of autophagosome formation. Evidently, autophagy is crucially involved in the regulation of the antiviral immune response. The striking correlation between SARS-CoV-2-induced aberrant inflammation and the observed autophagy defects [213] suggests that the virus-induced cytokine storm could be mediated by the failure of autophagy mechanisms to maintain cellular homeostasis.

**Figure 1:**
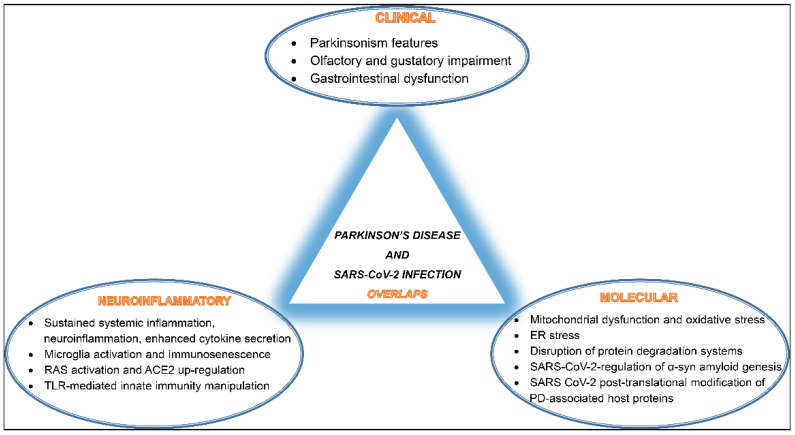
A schematic diagram of SARS-CoV-2 infection and Parkinson’s disease (PD) development overlaps listing shared clinical manifestations, common neuroinflammatory events, and mutually activated molecular pathways.

Overall, SARS-CoV-2 seems to interfere and disrupt several host cellular and molecular pathways involved in proper neuronal functions, potentially promoting PD pathogenesis. A summary of these overlaps is depicted in (Figure 1).

## 4. The Diverse Roles of Exosomes in Viral Infection and Neurogenerative Disease

### 4.1. Biogenesis of Exosomes

Exosomes are one of three main subtypes of extracellular vesicles (EVs) (microvesicles, exosomes, and apoptotic bodies) secreted by the cells [214]. They are secreted from the majority of cell types and can also be isolated from body fluids, such as saliva, plasma, serum, urine, CSF, etc. [215,216]. Exosomes contain cell-specific cargos of proteins, lipids, DNAs, and coding/non-coding RNAs from the donor cells that can be preferentially delivered to targeted recipient cells. They represent a recently discovered mode of intercellular communication that may play a major role in many cellular processes in both physiological and pathological conditions [217,218].

Exosomes are EVs [219] with a cup-shaped appearance in transmission electron microscopy and size ranging from 30 to 150 nm [220,221]. Exosomes emerge from the endosomal compartment following a series of events, starting with the inward budding of the plasma membrane that gives rise to the early endosome [222]. Then, budding and modification of the limiting membrane of endosomes leads to formation of intraluminal vesicles (ILVs), whose accumulation produces late endosomes or large multivesicular bodies (MVBs) [221,223]. MVBs can either fuse with the plasma membrane and release ILVs as exosomes into the extracellular space, or with lysosomes, where ILV cargo will be degraded [224]. Biogenesis of ILVs, trafficking of MVBs, and secretion of exosomes are mainly regulated by endosomal sorting complexes required for transport (ESCRT) [225]. The ESCRT machinery consists of a set of cytosolic protein complexes that become attracted to endosomes by membrane proteins usually tagged with ubiquitin [226]. This multi-subunit protein system is essential for membrane remodeling and cargo sorting of MVBs [227], although some studies found that ILVs are also formed in the absence of ESCRT machinery (ESCRT-independent pathway), with the enzyme sphingomyelinase and tetraspanin proteins, such as CD9, CD63, and CD81, playing a pivotal role [228]. When exosomes reach the target cell, they can be internalized through specific endocytotic mechanisms, fuse directly with the plasma membrane or bind to cellular surface receptors [229,230,231].

Exosomal surface indicators, such as the tetraspanins CD9, CD63, and CD81, ALG 2-interacting protein X (ALIX), tumor susceptibility gene 101 protein (TSG101), and ESCRT proteins, have all been identified and used to characterize exosomes in vitro and in vivo [31]. Because of their varied cellular origin, exosomal biomolecular composition is highly heterogeneous, bearing characteristics from both surface proteins and the cargo of their donor cells [223,232]. For example, neuron-derived exosomes are characterized by the presence of L1 cell adhesion molecule (L1CAM), a surface exosomal marker highly expressed in neurons [233]. Furthermore, their biocompatibility and bi-layered lipid structure, which shield cargo from degradation, reduce immunogenicity and enable exosomes to pass through major biological membranes, including the BBB [219]. Considering that exosomes can cross the BBB and act as fingerprints of their cellular originators, they could be used as CNS biomarkers that can be isolated and recovered from the periphery, with minimally invasive techniques [234].

### 4.2. Impact of Exosomes in PD Pathogenesis

The usage of exosomes for the removal of accumulated, misfolded proteins increases under pathological conditions, particularly in proteinopathies [235,236]. Specifically, when other cellular clearance systems, such as the proteasome and ALP, fail to eliminate aggregated amyloidogenic proteins, exosomes come to the rescue [237]. Thus, exosomes may have a prominent role in PD, as they represent a potential spreading pathway for misfolded proteins, thereby contributing to pathogenesis and also to disease diagnosis through cargo analysis [238,239].

The “prion-like” mode of α-syn spread lies at the heart of PD pathogenesis, as the molecular mechanisms leading to α-syn seeding and aggregation remain unknown [240]. Exosomes, as key intercellular mediators in the CNS, may provide a valuable vehicle for the transmission of α-syn [32,236]. Unlike other cell-to-cell transmission mechanisms, such as non-classical exocytosis or transport via nanotubules, exosomes can mediate α-syn transfer over longer distances [241]. The oligomeric form of α-syn is thought to be the toxic form causing neuronal death. Danzer and colleagues identified the presence of oligomeric α-syn in exosomes from continuous cell lines and primary cells and demonstrated that exosomal α-syn is more easily taken up by recipient cells than the free oligomeric form of protein [242]. Other researchers used various in vitro and ex vivo cellular systems to verify the presence of α-syn in exosomes [243,244]. Considering that loss of dopaminergic neurons is associated with PD progression and worsening of motor symptoms, exosomes generated by α-syn-treated microglia exhibited a considerably higher neuron apoptosis rate than control exosomes, in an in vitro experiment [245]. Exosomes isolated from patients with PD can induce the oligomerization of soluble α-syn in recipient cells, increasing neurotoxicity and speeding up α-syn aggregate formation [246]. Additionally, serum exosomes derived from PD patients contain α-syn, which can induce behavioral and pathological features of PD in mice [247]. Taken together, data from the abovementioned studies suggest that by regulating uptake and transfer of abnormal α-syn to nearby cells, exosomes are key regulators of PD pathogenesis and its spatiotemporal evolution [248,249].

Aside from trafficking α-syn, exosomes interfere in intercellular inflammatory pathways, enhancing the possibility of PD outcomes [250]. There is abundant evidence that neuroinflammation plays a vital role in PD onset and progression [23]. A sound inflammatory response is essential for tissue repair and misfolded protein breakdown, but an excessive and delayed inflammatory response can lead to a deregulated neuroinflammatory cycle [251]. Microglia are considered to be the resident brain macrophages. By phagocytosing dead cells and helping with the removal of misfolded protein aggregates from the brain, they play an important role in the removal of extracellular α-syn species, including exosome-contained protein [252]. At the same time, microglia could be activated by exosomal α-syn, and elicit an immunological response with the release of pro-inflammatory cytokines, resulting in dopaminergic neuron death [253]. Ιn addition to their well-established role in neuroinflammation, microglia appear to be involved in intercellular spreading of neurotoxic α-syn [254]. Furthermore, exogenous introduction of human α-syn preformed fibrils (PFFs) into primary microglial cell cultures stimulates the release of α-syn-containing exosomes, which were fully capable of inducing protein aggregation in recipient neurons [255]. This release of α-syn following PFF treatment could be an effort to monitor and control intracellular levels of misfolded protein, possibly attributed to the deregulation of the ALP pathway in microglia [255]. Autophagic activity impairment and lysosome dysfunction have been previously correlated to increased release of exosomes from neuronal cells and to exosomes-mediated α-syn spread and transmission [21,237,256].

Recent advances concerning immunosenescence studied its dissemination on tissue level, via exosomes. Specifically, cell-level stressors may induce a pro-inflammatory phenotypic shift in afflicted cells, and subsequently this shift may be communicated via specific exosomal cargo in a para- and juxtracrine manner [257]. Notably, interferon-responsive genes and proteins, such as the IFITM3 protein, may be loaded during such transmissions, representing a tissue-level signal of inflammation [258]. Depending on the cargo and cell of origin, exosomes may conversely ameliorate the SASP [259], thereby abating inflammation [260,261].

### 4.3. Exosomes as Biomarkers in PD Diagnosis

To date, the diagnosis of PD depends on the clinical manifestations of the disease and it is determined by the presence of motor symptoms [2]. PD diagnosis may be difficult, especially in early pre-symptomatic stages, due to the absence of motor symptoms [262]. Hence, a low clinical diagnostic accuracy rate in the preclinical phase has been reported [263]. The usage of exosomal cargo as a potential “biomarker” or “early indicator” of PD pathology and progression has piqued great interest. Notably, exosomal α-syn has been proposed as a potential biomarker for PD in multiple studies [238,239,246,264]. Shi and colleagues found that CNS-derived exosomal α-syn was considerably higher in PD patients than in controls and it had a significant relationship with disease severity [238]. Similarly, a longitudinal investigation revealed that α-syn levels in neuronal exosomes were significantly higher in patients with early-stage PD compared to control groups, and that higher α-syn levels were linked to the progression of motor impairment [264]. Another study found that levels of CNS-derived exosomal α-syn were lower in early-stage PD patients compared to individuals with essential tremor and the control group [239]. In salivary EVs from PD patients, it was demonstrated that absolute levels of α-syn oligomers and the ratio of α-syn oligomers over total α-syn were increased compared to healthy controls, indicating that they could be used as diagnostic biomarkers [265]. Mean levels of neuron-derived exosomal α-syn have been proposed to distinguish PD from other cases of atypical parkinsonism and neurodegenerative diseases, as a twofold increase was observed in patients with preclinical and established PD compared to the other etiologies [266].

As far as other neurospecific exosomal cargo proteins are concerned, it has been shown that CNS-derived exosomal tau levels were considerably higher in PD patients compared to controls, and notably but not significantly increased compared to those of AD patients [267]. Furthermore, levels of clusterin, apolipoprotein A1 (apoA1), and the complement C1r subcomponent were significantly lower in PD patients at Hoehn and Yar stages II and III (a scale for assessing the functional impairment caused by PD) compared to healthy controls [268]. Conversely, neuron-derived exosomal protein deglycase DJ-1 was higher in PD patients compared to controls [269].

In addition to misfolded proteins, exosomes serve as a conduit for transport of other RNA species such as microRNA (miRNA). Exosomal miRNAs regulate gene expression in recipient cells at a post-transcriptional level, thereby interfering with several physiological processes in the CNS, including homeostasis, neuron growth, cell migration, and brain endogenous immunity [270]. Mounting evidence has proposed a pivotal role for exosomal miRNAs in neurodegenerative diseases, including PD [271]. Firstly, they can inhibit protein synthesis after cellular uptake of exosomes [272]. Secondly, they can directly bind to TLRs and trigger neuroinflammation [273], and thirdly, they can induce oxidative stress pathways leading to neurotoxicity [274]. Given the diverse roles of miRNAs in PD pathogenesis, several studies have focused on their diagnostic value. Gui and colleagues studied the alterations in the miRNA profiles of CSF-derived exosomes from PD patients, finding that 16 exosomal miRNAs were elevated and 11 miRNAs were downregulated in PD in comparison with the control group. Characteristically, miR-1 and miR-19b-3p were identified to be considerably decreased in PD-CSF exosomes. MiR-153, miR-409-3p, miR-10a-5p, and let-7g-3p, on the other hand, were increased in that patient group [275]. Another study in serum exosomes from PD patients observed a drop in miR-19b expression and an elevation in miR-195/miR-24 expression in PD patients compared to healthy controls [276]. Finally, exosomal miRNAs were successfully used to differentially diagnose PD stages from healthy controls, showing that those miRNAs could serve as specific biomarkers both for early PD detection and PD progression [277].

### 4.4. Exosomes and SARS-CoV-2 Infection

Exosomes play a critical role in viral infections [278]. Exosomes and viruses share structural and physicochemical features, such as size, shape, biochemical composition, and biomolecule transportation pathways within cells [279,280]. Thus, exosomes constitute a new frontier in the realm of viral infections, including SARS-CoV-2.

A proposed way of viral spreading is via exosomes that contain viral particles or components. In the case of respiratory viruses, several studies showed that viral antigens are present in circulating exosomes recovered from lung transplant recipients infected with rhinovirus and respiratory syncytial virus [281]. In SARS-CoV-2 infection, exosomal cargo contains viral proteins or peptide fragments, such as the N and S proteins [282,283], whose presence has been linked to enhanced viral propagation, host immune reaction, and induction of a cytokine storm [284]. Barberis and colleagues identified for the first time viral genetic material in the exosomal cargo of COVID-19 patients, suggesting that SARS-CoV-2 may also be using the endocytic pathway to spread [285].

The “Trojan exosome hypothesis”, in which retroviruses employ EVs to penetrate host cells, boost viral propagation, and elude the immune response, was introduced by Gould and colleagues [286] and was later supported by several reports [40]. In SARS-CoV-2 infection, the virus uses multiple steps to achieve entry into the host cell, including ACE2-mediated receptor binding and transmembrane serine protease-2 (TMPRSS2)-mediated intracellular cleavage [287]. Recent data revealed that EVs carry and transfer ACE2 between different types of cells [288]. This led to the development of a competitive inhibitory therapy against SARS-CoV-2, in which ACE2-expressing EVs compete for the SARS-CoV-2 S protein S1 domain, limiting viral infection [289].

Exosomes released during viral infection contain inflammatory markers that cause a strong inflammatory response, acting as pathogen-related molecular patterns, thereby enhancing pathogenicity [290]. For example, exosomal CD9 has been shown to be involved in the route for EV-mediated viral transmission, by speeding up lentiviral infection and improving transduction effectiveness in B- and T-lymphocytes [291]. In addition, CD9 cooperates with TMPRSS2 to cleave viral fusion glycoproteins, thus facilitating the entry of coronaviruses, such as MERS-CoV, into lung cells [292]. These findings suggest that CD9 and other exosomal tetraspanins could facilitate SARS-CoV-2 infection. Proteomic analysis of plasma-derived exosomal cargo from COVID-19 patients revealed that circulating exosomal proteins are strongly correlated with pathological procedures leading to COVID-19 tissue damage, such as immune hyperactivation, coagulopathy induction, and inflammation [285]. Monosialodihexosyl ganglioside (GM3)-enriched exosomes are positively connected with disease severity in COVID-19 cases [293].

## 5. SARS-CoV-2-Related Εxosomal Cargo and Its Potential Roles in Post-COVID-19 Parkinsonism and PD Pathogenesis

Given the established role of exosomes both in pathophysiological neuronal processes, and PD development and progression [294,295], as well as their emerging significance in SARS-CoV-2 infection and propagation [285], we believe that cascading from SARS-CoV-2 infection to post-COVID-19 parkinsonism and, possibly PD onset via exosomal cargo, should be at the research forefront. The putative roles of SARS-CoV-2-related exosomal cargos in the development of viral parkinsonism and/or PD pathogenesis are outlined and described in the next two paragraphs.

### 5.1. Exosomal Cargo and Induction of Post-COVID-19 Neuroinflammation

Following COVID-19 recovery and viral clearance, exosomes could be at the center of neuroinflammatory crossroads. Experimental studies in other several systemic inflammatory conditions, such as obesity [296] and rheumatoid arthritis [297], have outlined a strong relationship between peripheral systemic inflammation and neuroinflammation [298,299]. Exosomes could operate as physical bridges between these two conditions since they can traverse the BBB. In vivo proof of exosomes acting as neuroinflammatory mediators under systemic inflammation conditions has recently been obtained. Specifically, in a mouse model that received serum-derived exosomes from lipopolysaccharide (LPS)-challenged mice, brain gliosis, CNS expression of pro-inflammatory cytokine mRNA and inflammation-associated miR-155 were all increased [300]. In addition, exosomes generated by peripheral immune cells, such as activated monocytes and macrophages, were shown to be taken up by neurons and astrocytes, resulting in pathological cargo dissemination and neurotoxicity [301].

Serum exosomes from PD patients revealed increasing levels of IL-1 and TNF-α inflammatory mediators in comparison with the control group, while intravenous or intrastriatal administration of PD exosomes to mice induced α-syn aggregation, microglia activation, and neurodegeneration of dopaminergic neurons, leading to worsening of motor symptoms [247]. Conversely, exosomes containing pro-inflammatory cytokines, such as IL-1, can effectively be shed by glial cells [302]. This glia-derived insidious exosomal cargo can spread to neurons, contributing to a vicious cycle of neuroinflammation and neurodegeneration [245], a phenomenon further associated with older age [303].

The presence of inflammatory mediators in exosomes derived from SARS-CoV-2 infected cells could potentially enhance the model of neuroinflammation and ensuing neurodegeneration via peripheral systemic inflammation, possibly promoting a cellular milieu that favors PD development and progression mechanisms. Exosomal analysis from COVID-19 patients revealed high levels of tenascin-C (TNC) and fibrinogen-β (FGB) compared to controls. Both TNC and FGB induce release of pro-inflammatory cytokines via NF-κB signaling, leading to the presence of TNF-α, IL-6, and chemokine CCL5 upon exposure of hepatocytes to exosomes from COVID-19 patients. In this regard, a potential “window” of inflammatory insults to distant tissues should be examined [304].

Current knowledge so far suggests that the SARS-CoV-2 S protein seems to play a central role in exosome-mediated regulation of neuroinflammatory events pertinent to neurodegeneration. Thus, S protein or S-derived fragments were discovered in plasma exosomes isolated from COVID-19 patients, with an increased exosomal presence in clinical cases with moderate rather than severe disease. Multiomics exosomal analysis identified several molecules involved in immune responses, inflammation, and activation of both coagulation and complement pathways in infected patients compared to the control group of healthy subjects [284].

Furthermore, an in vitro study demonstrated that ectopic expression of SARS-CoV-2 S protein in HEK-293T cells generated a large number of exosomes highly loaded with miR-148a and miR-590. These exosomal miRNAs decreased gene expression of ubiquitin specific peptidase 33 (USP33), a deubiquitinase enzyme (DUBs)-stabilizer of its target protein, and of interferon regulatory factor 9(IRF9). They also deregulated the USP33-IRF9 network following their internalization in human microglia cells. IRF9 had been previously identified as a protective functional protein in CNS homeostasis, and its absence could result in severe neurological damage in glial cultured cells due to interferon (IFN)-α-mediated overexpression of IFN-γ-like genes [305]. Reduced microglial USP and IRF9 levels effectively induced the production of important inflammatory gene pathways such as TNF-β, NF-κB, and IFN-β, culminating in neuroinflammatory cascade activation [306]. Apart from that, exosomes recovered from plasma of COVID-19 patients revealed the presence of S protein-derived fragments fully capable of inducing the immune system response. Evidently, exosomes from patients with mild disease severity exhibited higher amounts of MHC class II-antigen-presenting protein able to interact with CD4+ T-cells and boost their proliferation and activation [284]. MHC II overexpression, CD4 activation, and invasion of the CNS can all cause IFN-mediated phagocytic conversion of brain myeloid cells [307]. The ensuing neuroinflammation and dopaminergic neuron loss in the SNpc could conceivably lead to a PD phenotype [308,309].

Neuroinflammation mediated by host regulatory factors may also be conferred by SARS-CoV-2-manipulated exosomes. High-mobility group box 1 (HMGB1) is a nuclear protein involved in several CNS procedures such as inflammation, apoptosis, and autophagy regulation [310]. Elevated levels of HMGB1 in serum and CSF from PD patients have been observed [311]. Notably, inhibition of HMGB1 has been shown to reduce microglia-mediated neuroinflammation, neuron dopaminergic loss, and progression of PD pathology in PD animal models [311,312]. Neurofilament light chain (NfL) levels are a protein-indicator of axonal damage and serve as a biomarker for several neurodegenerative diseases, including PD [313,314]. At the same time, it has been suggested that NfL levels may potentially reflect neuroinflammatory processes leading to neurodegeneration in the early stages of multiple sclerosis development [315]. Analysis of the exosomal cargo of neuron-derived EVs (NDEV) isolated from post-COVID 19 patients with or without neurological symptoms showed high levels of HMGB1 and NfL compared to the control group, thereby implicating SARS-CoV-2 infection in the regulation of the two pro-PD development factors [316].

### 5.2. The Periphery-Exosomes-CNS Axis as a Promoter of Post-COVID-19 Parkinsonism and PD Development

Loaded with SARS-CoV-2 components and RNA, as well as virally-induced neuroregulatory molecules, exosomes are fully capable of accessing hard-to-reach neuroanatomical areas, such as the olfactory bulb [88], the hypothalamus [317], the DMV, and the brainstem [317,318,319]. A possible route allowing access to exosomes traveling long distances from peripheral tissues that are prime sites of infection, such as the intestine and the lungs, to the brain could be through retrograde axonal transport from peripheral nerves [320,321]. In that case, SARS-CoV-2-related exosomes could end up disrupting normal homeostatic molecular mechanisms in brain areas that have been previously associated with PD pathogenesis [322,323,324]. Indeed, a COVID-19 postmortem study detected major neurological damage, but only low levels of SARS-CoV-2 RNA in the brains of expired patients [325]. Furthermore, cortical accumulation of total α-syn was observed following viral eradication in a SARS-CoV-2 intranasally-infected hamster model without any indication of inflammation and neurodegeneration [326]. Extending the Braak hypothesis, one could argue that any neurological sequelae and neuropathological outcomes observed in COVID-19 survivors may emerge both directly, due to virus-exerted effects, and indirectly, through molecular and neuroinflammatory mediators, carried by SARS-CoV-2-related exosomes remaining in circulation even after elimination of the virus.

The neurological consequences of SARS-CoV-2 infection, according to Ahmed and colleagues, could be attributed, at least in part, to exosomal mRNA and transcriptional factors (Tfs) carried from the lungs to the brain areas [327]. These exosomal Tfs have the ability to regulate cellular gene expression transcriptionally and induce neuronal alterations consistent with imminent neurodegeneration. Among 19 exosomal Tfs found overexpressed during the acute phase of SARS-CoV-2 infection, BCL3, JUND, MXD1, IRF2, IRF9, and STAT1 were observed to activate genes associated with PD pathogenesis in strategic areas of the brain, such as the medial and lateral substantia nigra and the superior frontal gyrus region. These genes are implicated in a variety of physiological activities, including signal transduction, neuron death, and immunological surveillance. Evidently, their Tf-mediated dysregulation could contribute to neurodegeneration and PD pathology. For example, STAT1 triggers microglia activation and dopaminergic neurons’ autophagy under hypoxia, including COVID-19 manifested hypoxia [328,329,330].

Exosomes may be further implicated in the association between SARS-CoV-2 and PD via the transfer of protein expression regulators from the periphery, through the BBB and into the CNS. It has been suggested that such factors could interact with proteins that are highly expressed in the CNS and are linked to PD. A relevant study demonstrated that 24 host lung proteins were subjected to post-translational modifications by SARS-CoV-2 viral proteins. Then, they were taken up and transported out of the lungs into the CNS by exosomes, leading to local disruption of protein–protein interactions [163].

Finally, recent studies have verified the presence of SARS-CoV-2 viral proteins in brain-derived exosomes, potentially enhancing their role in SARS-CoV-2 propagation and pathogenicity. Indeed, cargo profiling of neuron-derived EVs (NDEV) and astrocyte-derived EVs (ADEV) recovered from the plasma of COVID-19 patients revealed significantly higher levels of critical SARS-CoV-2 S1 and N proteins, in all COVID-19 affected subgroups compared to controls. Notably, mean ADEV and NDEV levels of N protein could be used to distinguish the group of patients who developed long COVID-19 with neuropsychiatric manifestations from the long COVID-19 group without such complications and the recovered COVID-19 patients without long COVID-19 [283]. Furthermore, SARS-CoV-2 spike-derived fragments were found to be efficiently exhibited in exosomes from recovered patients who had previously suffered from both mild and severe COVID-19 [284].

In vitro investigations have demonstrated that DMV neurons are vulnerable to oxidative stress and that oxidative stress enhances intercellular α-syn propagation [331]. SARS-CoV-2 could exploit inflammatory exosomal cargo [306] to induce oxidative stress in the DMV, facilitate α-syn aggregation, and eventually promote post-COVID-19 parkinsonism through SARS-CoV-2 S and N proteins. This hypothesis could be supported by the fact that both viral proteins have been shown to increase total α-syn and phosphorylation at Ser129 (pS129) levels [332], speed up the ability of endogenous α-syn to form amyloid fibrils [167], and ultimately induce LB-like pathology [332].

Collectively, it is possible that the exosomal presence of these viral proteins illuminates connecting routes linking COVID-19 to PD. Given that SARS-CoV-2 viral components persist in exosomes during the acute and potentially the post-COVID-19 phase, one could reasonably hypothesize that the COVID-19-related exosomal cargo could act as a neurodegenerative promoter and probable elicitor of parkinsonism manifestation.

## 6. Conclusions

**Figure 2:**
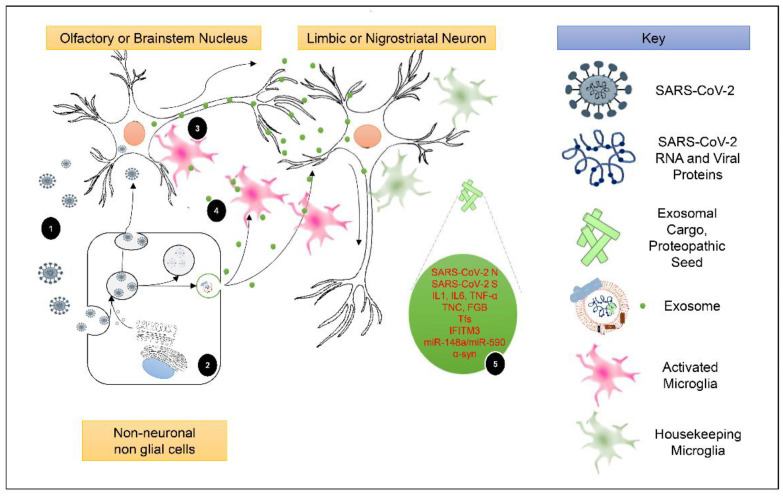
A hypothetical quasi-infectious model of CNS proteopathic seeding, with SARS-CoV-2 as the model virus. At step 1, SARS-CoV-2 neuroinvasion can occur either as a primary event by infecting neurons, or following infection of non-neuronal cells, such as brain endothelial cells, olfactory epithelial cells, or cells from peripheral infected tissues. Successful infection either of non-neuronal cells (2) or neurons (3) could lead to the production of exosomes that could be transmitted transynaptically (3), or intercellularly (4). The quasi-infectious concept indicates that the exosomal cargo is pathogenic and may be enhanced by the presence of viral components (5). SARS-CoV-2 S and N proteins could potentiate the formation of amyloid fibrils by endogenous α-syn in recipient cells. At the same time, exosomes could transmit α-syn to other brain cells in a “prion-like” mode. Exosomal immunomodulatory molecules could enhance neuroinflammatory processes in both neuronal and glia cells, while transcriptional regulators and miRNAs might activate intracellular signaling pathways and regulate gene transcription pertinent to neuroinflammation, oxidative stress, and other homeostatic cellular mechanisms. The ensuing microglial inflammatory phenotype turnover could enhance neuroinflammation further, resulting in enhanced neurodegeneration.

Although the role of viruses in PD pathogenesis is still debated, various studies have found a link between viruses and parkinsonism, suggesting that they may operate as an initiating trigger of primary PD or secondary parkinsonism. The outbreak of the COVID-19 pandemic led to the realization that elimination of the virus after recovery does not always signal the end of the disease, as many patients are burdened with post-COVID-19 manifestations. One of the great concerns of the medical community at this point is the likelihood that SARS-CoV-2 infection could lead to parkinsonism, a notion strongly supported by the PD-like symptoms observed in some patients during the acute or post-COVID-19 phase. Extending the hypothesis of post-COVID-19-induced parkinsonian phenotype, we present herein clinical, inflammatory, and molecular overlaps that have started to emerge and constitute a neurobiological connective network between these two diseases. Exosomes are key regulators of intercellular communication and have been shown to play a crucial role in neurodegenerative diseases. SARS-CoV-2 manipulates exosomes, altering their cargo and, as a result, their function, so that they may serve as a vehicle for viral spread. In doing so, these SARS-CoV-2-related exosomes may efficiently transport SARS-CoV-2 genetic material and viral proteins from the periphery, being the gut or lungs, or other tissues, in the CNS via BBB crossing. SARS-CoV-2 related exosomes, in particular, can potentially transmit SARS-CoV-2 fragments, transcriptional factors, and inflammatory mediators to brain cells, resulting in prolonged neuroinflammation and α-syn aggregation, which collectively contribute to PD development or a possible deterioration in people with genetic predisposition towards PD. Visualization of the hypothetical role of SARS-CoV-2-related exosomes and their cargo in PD pathogenesis is depicted in (Figure 2). In conclusion, we postulate in this review that cargo analysis of SARS-CoV-2-related exosomes, especially brain-derived ones, could serve as a compass for delineating underlying virus-mediated pro-PD development mechanisms and for detecting the much-dreaded post-COVID-19 parkinsonism storm. To this end, experimental and clinical studies will be conducted to validate our hypothesis.

## Data Availability

Not applicable.

## References

[1] Keener A.M., Bordelon Y.M. (2016). Parkinsonism. Proc. Semin. Neurol..

[2] Jankovic J. (2008). Parkinson’s disease: Clinical features and diagnosis. J. Neurol. Neurosurg. Psychiatry.

[3] Limphaibool N., Iwanowski P., Holstad M.J.V., Kobylarek D., Kozubski W. (2019). Infectious Etiologies of Parkinsonism: Pathomechanisms and Clinical Implications. Front. Neurol..

[4] Jang H., Boltz D.A., Webster R.G., Smeyne R.J. (2009). Viral parkinsonism. Biochim. Biophys. Acta.

[5] Kouli A., Torsney K.M., Kuan W.L., Stoker T.B., Greenland J.C. (2018). Parkinson’s Disease: Etiology, Neuropathology, and Pathogenesis. Parkinson’s Disease: Pathogenesis and Clinical Aspects.

[6] Lee A., Gilbert R.M. (2016). Epidemiology of Parkinson Disease. Neurol. Clin..

[7] Hirsch L., Jette N., Frolkis A., Steeves T., Pringsheim T. (2016). The Incidence of Parkinson’s Disease: A Systematic Review and Meta-Analysis. Neuroepidemiology.

[8] Twelves D., Perkins K.S., Counsell C. (2003). Systematic review of incidence studies of Parkinson’s disease. Mov. Disord. Off. J. Mov. Disord. Soc..

[9] Karimi-Moghadam A., Charsouei S., Bell B., Jabalameli M.R. (2018). Parkinson Disease from Mendelian Forms to Genetic Susceptibility: New Molecular Insights into the Neurodegeneration Process. Cell. Mol. Neurobiol..

[10] Warner T.T., Schapira A.H. (2003). Genetic and environmental factors in the cause of Parkinson’s disease. Ann. Neurol..

[11] Dauer W., Przedborski S. (2003). Parkinson’s disease: Mechanisms and models. Neuron.

[12] Khoo T.K., Yarnall A.J., Duncan G.W., Coleman S., O’Brien J.T., Brooks D.J., Barker R.A., Burn D.J. (2013). The spectrum of nonmotor symptoms in early Parkinson disease. Neurology.

[13] Pang S.Y., Ho P.W., Liu H.F., Leung C.T., Li L., Chang E.E.S., Ramsden D.B., Ho S.L. (2019). The interplay of aging, genetics and environmental factors in the pathogenesis of Parkinson’s disease. Transl. Neurodegener..

[14] Smeyne R.J., Noyce A.J., Byrne M., Savica R., Marras C. (2021). Infection and Risk of Parkinson’s Disease. J. Parkinson’s Dis..

[15] Baizabal-Carvallo J.F., Alonso-Juarez M. (2021). The role of viruses in the pathogenesis of Parkinson’s disease. Neural Regen. Res..

[16] Yamada T. (1996). Viral etiology of Parkinson’s disease: Focus on influenza A virus. Parkinsonism Relat. Disord..

[17] WHO Coronavirus Disease (COVID-19). https://www.who.int/emergencies/diseases/novel-coronavirus-2019.

[18] Brundin P., Nath A., Beckham J.D. (2020). Is COVID-19 a Perfect Storm for Parkinson’s Disease?. Trends Neurosci..

[19] Méndez-Guerrero A., Laespada-García M.I., Gómez-Grande A., Ruiz-Ortiz M., Blanco-Palmero V.A., Azcarate-Diaz F.J., Rábano-Suárez P., Álvarez-Torres E., de Fuenmayor-Fernández de la Hoz C.P., Vega Pérez D. (2020). Acute hypokinetic-rigid syndrome following SARS-CoV-2 infection. Neurology.

[20] Faber I., Brandão P.R.P., Menegatti F., de Carvalho Bispo D.D., Maluf F.B., Cardoso F. (2020). Coronavirus Disease 2019 and Parkinsonism: A Non-post-encephalitic Case. Mov. Disord. Off. J. Mov. Disord. Soc..

[21] Karabiyik C., Lee M.J., Rubinsztein D.C. (2017). Autophagy impairment in Parkinson’s disease. Essays Biochem..

[22] Dias V., Junn E., Mouradian M.M. (2013). The role of oxidative stress in Parkinson’s disease. J. Parkinson’s Dis..

[23] Hirsch E.C., Vyas S., Hunot S. (2012). Neuroinflammation in Parkinson’s disease. Parkinsonism Relat. Disord..

[24] Jucker M., Walker L.C. (2013). Self-propagation of pathogenic protein aggregates in neurodegenerative diseases. Nature.

[25] Guo J.L., Lee V.M. (2014). Cell-to-cell transmission of pathogenic proteins in neurodegenerative diseases. Nat. Med..

[26] Jan A., Gonçalves N.P., Vaegter C.B., Jensen P.H., Ferreira N. (2021). The Prion-Like Spreading of Alpha-Synuclein in Parkinson’s Disease: Update on Models and Hypotheses. Int. J. Mol. Sci..

[27] Visanji N.P., Brooks P.L., Hazrati L.N., Lang A.E. (2013). The prion hypothesis in Parkinson’s disease: Braak to the future. Acta Neuropathol. Commun..

[28] Recasens A., Dehay B., Bové J., Carballo-Carbajal I., Dovero S., Pérez-Villalba A., Fernagut P.O., Blesa J., Parent A., Perier C. (2014). Lewy body extracts from Parkinson disease brains trigger α-synuclein pathology and neurodegeneration in mice and monkeys. Ann. Neurol..

[29] Hansen C., Angot E., Bergström A.L., Steiner J.A., Pieri L., Paul G., Outeiro T.F., Melki R., Kallunki P., Fog K. (2011). α-Synuclein propagates from mouse brain to grafted dopaminergic neurons and seeds aggregation in cultured human cells. J. Clin. Investig..

[30] Volpicelli-Daley L.A., Luk K.C., Patel T.P., Tanik S.A., Riddle D.M., Stieber A., Meaney D.F., Trojanowski J.Q., Lee V.M. (2011). Exogenous α-synuclein fibrils induce Lewy body pathology leading to synaptic dysfunction and neuron death. Neuron.

[31] Théry C., Zitvogel L., Amigorena S. (2002). Exosomes: Composition, biogenesis and function. Nat. Rev. Immunol..

[32] Rastogi S., Sharma V., Bharti P.S., Rani K., Modi G.P., Nikolajeff F., Kumar S. (2021). The Evolving Landscape of Exosomes in Neurodegenerative Diseases: Exosomes Characteristics and a Promising Role in Early Diagnosis. Int. J. Mol. Sci..

[33] Gangoda L., Boukouris S., Liem M., Kalra H., Mathivanan S. (2015). Extracellular vesicles including exosomes are mediators of signal transduction: Are they protective or pathogenic?. Proteomics.

[34] Yuyama K., Igarashi Y. (2016). Physiological and pathological roles of exosomes in the nervous system. Biomol. Concepts.

[35] Caobi A., Nair M., Raymond A.D. (2020). Extracellular Vesicles in the Pathogenesis of Viral Infections in Humans. Viruses.

[36] Gurunathan S., Kang M.H., Kim J.H. (2021). Diverse Effects of Exosomes on COVID-19: A Perspective of Progress From Transmission to Therapeutic Developments. Front. Immunol..

[37] Karamichali E., Foka P., Valiakou V., Iliadis P., Loukaki D., Andresaki K., Papadopoulou G., Georgopoulou U., Koskinas J. (2022). Exosomal cargo as a key player of the immune response after direct-acting antiviral treatment in chronic hepatitis C patients. J. Hepatol..

[38] Yang L., Li J., Li S., Dang W., Xin S., Long S., Zhang W., Cao P., Lu J. (2021). Extracellular Vesicles Regulated by Viruses and Antiviral Strategies. Front. Cell Dev. Biol..

[39] Zhang L., Ju Y., Chen S., Ren L. (2021). Recent Progress on Exosomes in RNA Virus Infection. Viruses.

[40] Rezaie J., Aslan C., Ahmadi M., Zolbanin N.M., Kashanchi F., Jafari R. (2021). The versatile role of exosomes in human retroviral infections: From immunopathogenesis to clinical application. Cell Biosci..

[41] McCall S., Vilensky J.A., Gilman S., Taubenberger J.K. (2008). The relationship between encephalitis lethargica and influenza: A critical analysis. J. Neurovirology.

[42] Wijarnpreecha K., Chesdachai S., Jaruvongvanich V., Ungprasert P. (2018). Hepatitis C virus infection and risk of Parkinson’s disease: A systematic review and meta-analysis. Eur. J. Gastroenterol. Hepatol..

[43] Marttila R.J., Rinne U.K. (1978). Herpes simplex virus antibodies in patients with Parkinson’s disease. J. Neurol. Sci..

[44] Mirsattari S.M., Power C., Nath A. (1998). Parkinsonism with HIV infection. Mov. Disord. Off. J. Mov. Disord. Soc..

[45] Lai S.W., Lin C.H., Lin H.F., Lin C.L., Lin C.C., Liao K.F. (2017). Herpes zoster correlates with increased risk of Parkinson’s disease in older people: A population-based cohort study in Taiwan. Medicine.

[46] Robinson R.L., Shahida S., Madan N., Rao S., Khardori N. (2003). Transient parkinsonism in West Nile virus encephalitis. Am. J. Med..

[47] Murgod U.A., Muthane U.B., Ravi V., Radhesh S., Desai A. (2001). Persistent movement disorders following Japanese encephalitis. Neurology.

[48] Das K., Ghosh M., Nag C., Nandy S.P., Banerjee M., Datta M., Devi G., Chaterjee G. (2011). Role of familial, environmental and occupational factors in the development of Parkinson’s disease. Neuro-Degener. Dis..

[49] Espay A.J., Henderson K.K. (2011). Postencephalitic parkinsonism and basal ganglia necrosis due to Epstein-Barr virus infection. Neurology.

[50] Toovey S., Jick S.S., Meier C.R. (2011). Parkinson’s disease or Parkinson symptoms following seasonal influenza. Influenza Other Respir. Viruses.

[51] Cocoros N.M., Svensson E., Szépligeti S.K., Vestergaard S.V., Szentkúti P., Thomsen R.W., Borghammer P., Sørensen H.T., Henderson V.W. (2021). Long-term Risk of Parkinson Disease Following Influenza and Other Infections. JAMA Neurol..

[52] Rohn T.T., Catlin L.W. (2011). Immunolocalization of influenza A virus and markers of inflammation in the human Parkinson’s disease brain. PLoS ONE.

[53] Jang H., Boltz D., Sturm-Ramirez K., Shepherd K.R., Jiao Y., Webster R., Smeyne R.J. (2009). Highly pathogenic H5N1 influenza virus can enter the central nervous system and induce neuroinflammation and neurodegeneration. Proc. Natl. Acad. Sci. USA.

[54] Sadasivan S., Zanin M., O’Brien K., Schultz-Cherry S., Smeyne R.J. (2015). Induction of microglia activation after infection with the non-neurotropic A/CA/04/2009 H1N1 influenza virus. PLoS ONE.

[55] Marreiros R., Müller-Schiffmann A., Trossbach S.V., Prikulis I., Hänsch S., Weidtkamp-Peters S., Moreira A.R., Sahu S., Soloviev I., Selvarajah S. (2020). Disruption of cellular proteostasis by H1N1 influenza A virus causes α-synuclein aggregation. Proc. Natl. Acad. Sci. USA.

[56] Kasen A., Houck C., Burmeister A.R., Sha Q., Brundin L., Brundin P. (2022). Upregulation of α-synuclein following immune activation: Possible trigger of Parkinson’s disease. Neurobiol. Dis..

[57] Beatman E.L., Massey A., Shives K.D., Burrack K.S., Chamanian M., Morrison T.E., Beckham J.D. (2015). Alpha-Synuclein Expression Restricts RNA Viral Infections in the Brain. J. Virol..

[58] Caggiu E., Paulus K., Arru G., Piredda R., Sechi G.P., Sechi L.A. (2016). Humoral cross reactivity between α-synuclein and herpes simplex-1 epitope in Parkinson’s disease, a triggering role in the disease?. J. Neuroimmunol..

[59] Caggiu E., Paulus K., Galleri G., Arru G., Manetti R., Sechi G.P., Sechi L.A. (2017). Homologous HSV1 and alpha-synuclein peptides stimulate a T cell response in Parkinson’s disease. J. Neuroimmunol..

[60] Fazzini E., Fleming J., Fahn S. (1992). Cerebrospinal fluid antibodies to coronavirus in patients with Parkinson’s disease. Mov. Disord. Off. J. Mov. Disord. Soc..

[61] Almaghaslah D., Kandasamy G., Almanasef M., Vasudevan R., Chandramohan S. (2020). Review on the coronavirus disease (COVID-19) pandemic: Its outbreak and current status. Int. J. Clin. Pract..

[62] Wu J.T., Leung K., Leung G.M. (2020). Nowcasting and forecasting the potential domestic and international spread of the 2019-nCoV outbreak originating in Wuhan, China: A modelling study. Lancet.

[63] Hui D.S., Azhar E.I., Madani T.A., Ntoumi F., Kock R., Dar O., Ippolito G., McHugh T.D., Memish Z.A., Drosten C. (2020). The continuing 2019-nCoV epidemic threat of novel coronaviruses to global health-The latest 2019 novel coronavirus outbreak in Wuhan, China. Int. J. Infect. Dis. IJID Off. Publ. Int. Soc. Infect. Dis..

[64] Johansson M.A., Quandelacy T.M., Kada S., Prasad P.V., Steele M., Brooks J.T., Slayton R.B., Biggerstaff M., Butler J.C. (2021). SARS-CoV-2 Transmission From People Without COVID-19 Symptoms. JAMA Netw. Open.

[65] Zhou L., Ayeh S.K., Chidambaram V., Karakousis P.C. (2021). Modes of transmission of SARS-CoV-2 and evidence for preventive behavioral interventions. BMC Infect. Dis..

[66] Harapan B.N., Yoo H.J. (2021). Neurological symptoms, manifestations, and complications associated with severe acute respiratory syndrome coronavirus 2 (SARS-CoV-2) and coronavirus disease 19 (COVID-19). J. Neurol..

[67] Paterson R.W., Brown R.L., Benjamin L., Nortley R., Wiethoff S., Bharucha T., Jayaseelan D.L., Kumar G., Raftopoulos R.E., Zambreanu L. (2020). The emerging spectrum of COVID-19 neurology: Clinical, radiological and laboratory findings. Brain J. Neurol..

[68] Beghi E., Giussani G., Westenberg E., Allegri R., Garcia-Azorin D., Guekht A., Frontera J., Kivipelto M., Mangialasche F., Mukaetova-Ladinska E.B. (2022). Acute and post-acute neurological manifestations of COVID-19: Present findings, critical appraisal, and future directions. J. Neurol..

[69] Taquet M., Dercon Q., Luciano S., Geddes J.R., Husain M., Harrison P.J. (2021). Incidence, co-occurrence, and evolution of long-COVID features: A 6-month retrospective cohort study of 273,618 survivors of COVID-19. PLoS Med..

[70] National Institute for Health and Care Excellence (NICE) (2020). National Institute for Health and Care Excellence: Clinical Guidelines. COVID-19 Rapid Guideline: Managing the Long-Term Effects of COVID-19.

[71] Stephenson J. (2021). New Federal Guidance Says COVID-19’s Long-term Effects Can Qualify as a Disability. JAMA Health Forum.

[72] Mehandru S., Merad M. (2022). Pathological sequelae of long-haul COVID. Nat. Immunol..

[73] Camargo-Martínez W., Lozada-Martínez I., Escobar-Collazos A., Navarro-Coronado A., Moscote-Salazar L., Pacheco-Hernández A., Janjua T., Bosque-Varela P. (2021). Post-COVID 19 neurological syndrome: Implications for sequelae’s treatment. J. Clin. Neurosci. Off. J. Neurosurg. Soc. Australas..

[74] Shah W., Hillman T., Playford E.D., Hishmeh L. (2021). Managing the long term effects of covid-19: Summary of NICE, SIGN, and RCGP rapid guideline. BMJ.

[75] Khorramdelazad H., Kazemi M.H., Najafi A., Keykhaee M., Zolfaghari Emameh R., Falak R. (2021). Immunopathological similarities between COVID-19 and influenza: Investigating the consequences of Co-infection. Microb. Pathog..

[76] Cohen M.E., Eichel R., Steiner-Birmanns B., Janah A., Ioshpa M., Bar-Shalom R., Paul J.J., Gaber H., Skrahina V., Bornstein N.M. (2020). A case of probable Parkinson’s disease after SARS-CoV-2 infection. Lancet Neurol..

[77] Rao A.R., Hidayathullah S.M., Hegde K., Adhikari P. (2022). Parkinsonism: An emerging post COVID sequelae. IDCases.

[78] Boura I., Chaudhuri K.R. (2022). Coronavirus Disease 2019 and Related Parkinsonism: The Clinical Evidence Thus Far. Mov. Disord. Clin. Pract..

[79] Cilia R., Bonvegna S., Straccia G., Andreasi N.G., Elia A.E., Romito L.M., Devigili G., Cereda E., Eleopra R. (2020). Effects of COVID-19 on Parkinson’s Disease Clinical Features: A Community-Based Case-Control Study. Mov. Disord. Off. J. Mov. Disord. Soc..

[80] Brown E.G., Chahine L.M., Goldman S.M., Korell M., Mann E., Kinel D.R., Arnedo V., Marek K.L., Tanner C.M. (2020). The Effect of the COVID-19 Pandemic on People with Parkinson’s Disease. J. Parkinson’s Dis..

[81] Montenegro P., Moral I., Puy A., Cordero E., Chantada N., Cuixart L., Brotons C. (2022). Prevalence of Post COVID-19 Condition in Primary Care: A Cross Sectional Study. Int. J. Environ. Res. Public Health.

[82] Nasserie T., Hittle M., Goodman S.N. (2021). Assessment of the Frequency and Variety of Persistent Symptoms Among Patients With COVID-19: A Systematic Review. JAMA Netw. Open.

[83] Leta V., Rodríguez-Violante M., Abundes A., Rukavina K., Teo J.T., Falup-Pecurariu C., Irincu L., Rota S., Bhidayasiri R., Storch A. (2021). Parkinson’s Disease and Post-COVID-19 Syndrome: The Parkinson’s Long-COVID Spectrum. Mov. Disord. Off. J. Mov. Disord. Soc..

[84] Doty R.L. (2012). Olfactory dysfunction in Parkinson disease. Nat. Rev. Neurol..

[85] Tarakad A., Jankovic J. (2017). Anosmia and Ageusia in Parkinson’s Disease. Int. Rev. Neurobiol..

[86] Augustin M., Schommers P., Stecher M., Dewald F., Gieselmann L., Gruell H., Horn C., Vanshylla K., Cristanziano V.D., Osebold L. (2021). Post-COVID syndrome in non-hospitalised patients with COVID-19: A longitudinal prospective cohort study. Lancet Reg. Health. Eur..

[87] Vavougios G.D. (2020). Potentially irreversible olfactory and gustatory impairments in COVID-19: Indolent vs. fulminant SARS-CoV-2 neuroinfection. Brain Behav. Immun..

[88] Meinhardt J., Radke J., Dittmayer C., Franz J., Thomas C., Mothes R., Laue M., Schneider J., Brünink S., Greuel S. (2021). Olfactory transmucosal SARS-CoV-2 invasion as a port of central nervous system entry in individuals with COVID-19. Nat. Neurosci..

[89] Brann D.H., Tsukahara T., Weinreb C., Lipovsek M., Van den Berge K., Gong B., Chance R., Macaulay I.C., Chou H.J., Fletcher R.B. (2020). Non-neuronal expression of SARS-CoV-2 entry genes in the olfactory system suggests mechanisms underlying COVID-19-associated anosmia. Sci. Adv..

[90] Hubbard P.S., Esiri M.M., Reading M., McShane R., Nagy Z. (2007). Alpha-synuclein pathology in the olfactory pathways of dementia patients. J. Anat..

[91] Braak H., Del Tredici K., Rüb U., de Vos R.A., Jansen Steur E.N., Braak E. (2003). Staging of brain pathology related to sporadic Parkinson’s disease. Neurobiol. Aging.

[92] Braak H., Del Tredici K. (2017). Neuropathological Staging of Brain Pathology in Sporadic Parkinson’s disease: Separating the Wheat from the Chaff. J. Parkinson’s Dis..

[93] Beach T.G., White C.L., Hladik C.L., Sabbagh M.N., Connor D.J., Shill H.A., Sue L.I., Sasse J., Bachalakuri J., Henry-Watson J. (2009). Olfactory bulb alpha-synucleinopathy has high specificity and sensitivity for Lewy body disorders. Acta Neuropathol..

[94] Cersosimo M.G., Raina G.B., Pecci C., Pellene A., Calandra C.R., Gutiérrez C., Micheli F.E., Benarroch E.E. (2013). Gastrointestinal manifestations in Parkinson’s disease: Prevalence and occurrence before motor symptoms. J. Neurol..

[95] Devos D., Lebouvier T., Lardeux B., Biraud M., Rouaud T., Pouclet H., Coron E., Bruley des Varannes S., Naveilhan P., Nguyen J.M. (2013). Colonic inflammation in Parkinson’s disease. Neurobiol. Dis..

[96] Huang Y., Liao J., Liu X., Zhong Y., Cai X., Long L. (2021). Review: The Role of Intestinal Dysbiosis in Parkinson’s Disease. Front. Cell. Infect. Microbiol..

[97] Klingelhoefer L., Reichmann H. (2015). Pathogenesis of Parkinson disease--the gut-brain axis and environmental factors. Nat. Rev. Neurol..

[98] Shannon K.M., Keshavarzian A., Dodiya H.B., Jakate S., Kordower J.H. (2012). Is alpha-synuclein in the colon a biomarker for premotor Parkinson’s disease? Evidence from 3 cases. Mov. Disord. Off. J. Mov. Disord. Soc..

[99] Braak H., Rüb U., Gai W.P., Del Tredici K. (2003). Idiopathic Parkinson’s disease: Possible routes by which vulnerable neuronal types may be subject to neuroinvasion by an unknown pathogen. J. Neural Transm..

[100] Anselmi L., Toti L., Bove C., Hampton J., Travagli R.A. (2017). A Nigro-Vagal Pathway Controls Gastric Motility and Is Affected in a Rat Model of Parkinsonism. Gastroenterology.

[101] Beach T.G., Adler C.H., Sue L.I., Vedders L., Lue L., White Iii C.L., Akiyama H., Caviness J.N., Shill H.A., Sabbagh M.N. (2010). Multi-organ distribution of phosphorylated alpha-synuclein histopathology in subjects with Lewy body disorders. Acta Neuropathol..

[102] Gelpi E., Navarro-Otano J., Tolosa E., Gaig C., Compta Y., Rey M.J., Martí M.J., Hernández I., Valldeoriola F., Reñé R. (2014). Multiple organ involvement by alpha-synuclein pathology in Lewy body disorders. Mov. Disord. Off. J. Mov. Disord. Soc..

[103] Kim S., Kwon S.H., Kam T.I., Panicker N., Karuppagounder S.S., Lee S., Lee J.H., Kim W.R., Kook M., Foss C.A. (2019). Transneuronal Propagation of Pathologic α-Synuclein from the Gut to the Brain Models Parkinson’s Disease. Neuron.

[104] Chaves Andrade M., Souza de Faria R., Avelino Mota Nobre S. (2020). COVID-19: Can the symptomatic SARS-CoV-2 infection affect the homeostasis of the gut-brain-microbiota axis?. Med. Hypotheses.

[105] Xu J., Wu Z., Zhang M., Liu S., Zhou L., Yang C., Liu C. (2021). The Role of the Gastrointestinal System in Neuroinvasion by SARS-CoV-2. Front. Neurosci..

[106] Tao W., Wang X., Zhang G., Guo M., Ma H., Zhao D., Sun Y., He J., Liu L., Zhang K. (2021). Re-detectable positive SARS-CoV-2 RNA tests in patients who recovered from COVID-19 with intestinal infection. Protein Cell.

[107] Kaźmierczak-Siedlecka K., Vitale E., Makarewicz W. (2020). COVID-19-gastrointestinal and gut microbiota-related aspects. Eur. Rev. Med. Pharmacol. Sci..

[108] Viana S.D., Nunes S., Reis F. (2020). ACE2 imbalance as a key player for the poor outcomes in COVID-19 patients with age-related comorbidities-Role of gut microbiota dysbiosis. Ageing Res. Rev..

[109] Mohajeri M.H., La Fata G., Steinert R.E., Weber P. (2018). Relationship between the gut microbiome and brain function. Nutr. Rev..

[110] Sharon G., Sampson T.R., Geschwind D.H., Mazmanian S.K. (2016). The Central Nervous System and the Gut Microbiome. Cell.

[111] Troncoso-Escudero P., Parra A., Nassif M., Vidal R.L. (2018). Outside in: Unraveling the Role of Neuroinflammation in the Progression of Parkinson’s Disease. Front. Neurol..

[112] Marogianni C., Sokratous M., Dardiotis E., Hadjigeorgiou G.M., Bogdanos D., Xiromerisiou G. (2020). Neurodegeneration and Inflammation-An Interesting Interplay in Parkinson’s Disease. Int. J. Mol. Sci..

[113] Majde J.A. (2010). Neuroinflammation resulting from covert brain invasion by common viruses-a potential role in local and global neurodegeneration. Med. Hypotheses.

[114] Almutairi M.M., Sivandzade F., Albekairi T.H., Alqahtani F., Cucullo L. (2021). Neuroinflammation and Its Impact on the Pathogenesis of COVID-19. Front. Med..

[115] Bauer L., Laksono B.M., de Vrij F.M.S., Kushner S.A., Harschnitz O., van Riel D. (2022). The neuroinvasiveness, neurotropism, and neurovirulence of SARS-CoV-2. Trends Neurosci..

[116] Yachou Y., El Idrissi A., Belapasov V., Ait Benali S. (2020). Neuroinvasion, neurotropic, and neuroinflammatory events of SARS-CoV-2: Understanding the neurological manifestations in COVID-19 patients. Neurol. Sci. Off. J. Ital. Neurol. Soc. Ital. Soc. Clin. Neurophysiol..

[117] Cárdenas G., Fragoso G., Sciutto E. (2022). Neuroinflammation in Severe Acute Respiratory Syndrome Coronavirus-2 (SARS-CoV-2) infection: Pathogenesis and clinical manifestations. Curr. Opin. Pharmacol..

[118] Rowaiye A.B., Okpalefe O.A., Onuh Adejoke O., Ogidigo J.O., Hannah Oladipo O., Ogu A.C., Oli A.N., Olofinase S., Onyekwere O., Rabiu Abubakar A. (2021). Attenuating the Effects of Novel COVID-19 (SARS-CoV-2) Infection-Induced Cytokine Storm and the Implications. J. Inflamm. Res..

[119] Olbei M., Hautefort I., Modos D., Treveil A., Poletti M., Gul L., Shannon-Lowe C.D., Korcsmaros T. (2021). SARS-CoV-2 Causes a Different Cytokine Response Compared to Other Cytokine Storm-Causing Respiratory Viruses in Severely Ill Patients. Front. Immunol..

[120] Islam H., Chamberlain T.C., Mui A.L., Little J.P. (2021). Elevated Interleukin-10 Levels in COVID-19: Potentiation of Pro-Inflammatory Responses or Impaired Anti-Inflammatory Action?. Front. Immunol..

[121] Santa Cruz A., Mendes-Frias A., Oliveira A.I., Dias L., Matos A.R., Carvalho A., Capela C., Pedrosa J., Castro A.G., Silvestre R. (2021). Interleukin-6 Is a Biomarker for the Development of Fatal Severe Acute Respiratory Syndrome Coronavirus 2 Pneumonia. Front. Immunol..

[122] Lu Q., Zhu Z., Tan C., Zhou H., Hu Y., Shen G., Zhu P., Yang G., Xie X. (2021). Changes of serum IL-10, IL-1β, IL-6, MCP-1, TNF-α, IP-10 and IL-4 in COVID-19 patients. Int. J. Clin. Pract..

[123] Mehta P., Fajgenbaum D.C. (2021). Is severe COVID-19 a cytokine storm syndrome: A hyperinflammatory debate. Curr. Opin. Rheumatol..

[124] Chen H., O’Reilly E.J., Schwarzschild M.A., Ascherio A. (2008). Peripheral inflammatory biomarkers and risk of Parkinson’s disease. Am. J. Epidemiol..

[125] Hsu R.J., Yu W.C., Peng G.R., Ye C.H., Hu S., Chong P.C.T., Yap K.Y., Lee J.Y.C., Lin W.C., Yu S.H. (2022). The Role of Cytokines and Chemokines in Severe Acute Respiratory Syndrome Coronavirus 2 Infections. Front. Immunol..

[126] Kadry H., Noorani B., Cucullo L. (2020). A blood-brain barrier overview on structure, function, impairment, and biomarkers of integrity. Fluids Barriers CNS.

[127] Hawkins B.T., Davis T.P. (2005). The blood-brain barrier/neurovascular unit in health and disease. Pharmacol. Rev..

[128] Yu X., Ji C., Shao A. (2020). Neurovascular Unit Dysfunction and Neurodegenerative Disorders. Front. Neurosci..

[129] Klein R.S., Garber C., Funk K.E., Salimi H., Soung A., Kanmogne M., Manivasagam S., Agner S., Cain M. (2019). Neuroinflammation During RNA Viral Infections. Annu. Rev. Immunol..

[130] Yang R.C., Huang K., Zhang H.P., Li L., Zhang Y.F., Tan C., Chen H.C., Jin M.L., Wang X.R. (2022). SARS-CoV-2 productively infects human brain microvascular endothelial cells. J. Neuroinflamm..

[131] Raghavan S., Kenchappa D.B., Leo M.D. (2021). SARS-CoV-2 Spike Protein Induces Degradation of Junctional Proteins That Maintain Endothelial Barrier Integrity. Front. Cardiovasc. Med..

[132] Reynolds J.L., Mahajan S.D. (2021). SARS-COV2 Alters Blood Brain Barrier Integrity Contributing to Neuro-Inflammation. J. Neuroimmune Pharmacol. Off. J. Soc. NeuroImmune Pharmacol..

[133] John G.R., Lee S.C., Brosnan C.F. (2003). Cytokines: Powerful regulators of glial cell activation. Neurosci. A Rev. J. Bringing Neurobiol. Neurol. Psychiatry.

[134] da Fonseca A.C., Matias D., Garcia C., Amaral R., Geraldo L.H., Freitas C., Lima F.R. (2014). The impact of microglial activation on blood-brain barrier in brain diseases. Front. Cell. Neurosci..

[135] Rama Rao K.V., Kielian T. (2015). Neuron-astrocyte interactions in neurodegenerative diseases: Role of neuroinflammation. Clin. Exp. Neuroimmunol..

[136] Liddelow S.A., Guttenplan K.A., Clarke L.E., Bennett F.C., Bohlen C.J., Schirmer L., Bennett M.L., Münch A.E., Chung W.S., Peterson T.C. (2017). Neurotoxic reactive astrocytes are induced by activated microglia. Nature.

[137] Koprich J.B., Reske-Nielsen C., Mithal P., Isacson O. (2008). Neuroinflammation mediated by IL-1beta increases susceptibility of dopamine neurons to degeneration in an animal model of Parkinson’s disease. J. Neuroinflamm..

[138] Frank M.G., Nguyen K.H., Ball J.B., Hopkins S., Kelley T., Baratta M.V., Fleshner M., Maier S.F. (2022). SARS-CoV-2 spike S1 subunit induces neuroinflammatory, microglial and behavioral sickness responses: Evidence of PAMP-like properties. Brain Behav. Immun..

[139] Chaudhry Z.L., Klenja D., Janjua N., Cami-Kobeci G., Ahmed B.Y. (2020). COVID-19 and Parkinson’s Disease: Shared Inflammatory Pathways Under Oxidative Stress. Brain Sci..

[140] Kumar D., Jahan S., Khan A., Siddiqui A.J., Redhu N.S., Wahajuddin, Khan J., Banwas S., Alshehri B., Alaidarous M. (2021). Neurological Manifestation of SARS-CoV-2 Induced Inflammation and Possible Therapeutic Strategies Against COVID-19. Mol. Neurobiol..

[141] Sahu M.R., Rani L., Subba R., Mondal A.C. (2022). Cellular senescence in the aging brain: A promising target for neurodegenerative diseases. Mech. Ageing Dev..

[142] Martínez-Cué C., Rueda N. (2020). Cellular Senescence in Neurodegenerative Diseases. Front. Cell. Neurosci..

[143] Tripathi U., Nchioua R., Prata L., Zhu Y., Gerdes E.O.W., Giorgadze N., Pirtskhalava T., Parker E., Xue A., Espindola-Netto J.M. (2021). SARS-CoV-2 causes senescence in human cells and exacerbates the senescence-associated secretory phenotype through TLR-3. Aging.

[144] Kandhaya-Pillai R., Yang X., Tchkonia T., Martin G.M., Kirkland J.L., Oshima J. (2022). TNF-α/IFN-γ synergy amplifies senescence-associated inflammation and SARS-CoV-2 receptor expression via hyper-activated JAK/STAT1. Aging Cell.

[145] Müller L., Di Benedetto S. (2021). How Immunosenescence and Inflammaging May Contribute to Hyperinflammatory Syndrome in COVID-19. Int. J. Mol. Sci..

[146] Matschke J., Lütgehetmann M., Hagel C., Sperhake J.P., Schröder A.S., Edler C., Mushumba H., Fitzek A., Allweiss L., Dandri M. (2020). Neuropathology of patients with COVID-19 in Germany: A post-mortem case series. Lancet Neurol..

[147] Schurink B., Roos E., Radonic T., Barbe E., Bouman C.S.C., de Boer H.H., de Bree G.J., Bulle E.B., Aronica E.M., Florquin S. (2020). Viral presence and immunopathology in patients with lethal COVID-19: A prospective autopsy cohort study. Lancet Microbe.

[148] Yang A.C., Kern F., Losada P.M., Agam M.R., Maat C.A., Schmartz G.P., Fehlmann T., Stein J.A., Schaum N., Lee D.P. (2021). Dysregulation of brain and choroid plexus cell types in severe COVID-19. Nature.

[149] Vavougios G.D., Breza M., Mavridis T., Krogfelt K.A. (2021). FYN, SARS-CoV-2, and IFITM3 in the neurobiology of Alzheimer’s disease. Brain Disord..

[150] Clairembault T., Kamphuis W., Leclair-Visonneau L., Rolli-Derkinderen M., Coron E., Neunlist M., Hol E.M., Derkinderen P. (2014). Enteric GFAP expression and phosphorylation in Parkinson’s disease. J. Neurochem..

[151] Rodriguez-Perez A.I., Garrido-Gil P., Pedrosa M.A., Garcia-Garrote M., Valenzuela R., Navarro G., Franco R., Labandeira-Garcia J.L. (2020). Angiotensin type 2 receptors: Role in aging and neuroinflammation in the substantia nigra. Brain Behav. Immun..

[152] Paul D., Mohankumar S.K., Thomas R.S., Kheng C.B., Basavan D. (2022). Potential Implications of Angiotensin-converting Enzyme 2 Blockades on Neuroinflammation in SARS-CoV-2 Infection. Curr. Drug Targets.

[153] Williams A., Branscome H., Khatkar P., Mensah G.A., Al Sharif S., Pinto D.O., DeMarino C., Kashanchi F. (2021). A comprehensive review of COVID-19 biology, diagnostics, therapeutics, and disease impacting the central nervous system. J. Neurovirol..

[154] Pavel A., Murray D.K., Stoessl A.J. (2020). COVID-19 and selective vulnerability to Parkinson’s disease. Lancet Neurol..

[155] Klingenstein M., Klingenstein S., Neckel P.H., Mack A.F., Wagner A.P., Kleger A., Liebau S., Milazzo A. (2020). Evidence of SARS-CoV2 Entry Protein ACE2 in the Human Nose and Olfactory Bulb. Cells Tissues Organs.

[156] Chen R., Wang K., Yu J., Howard D., French L., Chen Z., Wen C., Xu Z. (2020). The Spatial and Cell-Type Distribution of SARS-CoV-2 Receptor ACE2 in the Human and Mouse Brains. Front. Neurol..

[157] Wan D., Du T., Hong W., Chen L., Que H., Lu S., Peng X. (2021). Neurological complications and infection mechanism of SARS-COV-2. Signal Transduct. Target. Ther..

[158] Yang L., Han Y., Nilsson-Payant B.E., Gupta V., Wang P., Duan X., Tang X., Zhu J., Zhao Z., Jaffré F. (2020). A Human Pluripotent Stem Cell-based Platform to Study SARS-CoV-2 Tropism and Model Virus Infection in Human Cells and Organoids. Cell Stem Cell.

[159] Aboudounya M.M., Heads R.J. (2021). COVID-19 and Toll-Like Receptor 4 (TLR4): SARS-CoV-2 May Bind and Activate TLR4 to Increase ACE2 Expression, Facilitating Entry and Causing Hyperinflammation. Mediat. Inflamm..

[160] Kawasaki T., Kawai T. (2014). Toll-like receptor signaling pathways. Front. Immunol..

[161] Conte C. (2021). Possible Link between SARS-CoV-2 Infection and Parkinson’s Disease: The Role of Toll-Like Receptor 4. Int. J. Mol. Sci..

[162] Lecours C., Bordeleau M., Cantin L., Parent M., Paolo T.D., Tremblay M. (2018). Microglial Implication in Parkinson’s Disease: Loss of Beneficial Physiological Roles or Gain of Inflammatory Functions?. Front. Cell. Neurosci..

[163] Estrada E. (2021). Cascading from SARS-CoV-2 to Parkinson’s Disease through Protein-Protein Interactions. Viruses.

[164] Wen H., Zhan L., Chen S., Long L., Xu E. (2017). Rab7 may be a novel therapeutic target for neurologic diseases as a key regulator in autophagy. J. Neurosci. Res..

[165] Shin W.H., Park J.H., Chung K.C. (2020). The central regulator p62 between ubiquitin proteasome system and autophagy and its role in the mitophagy and Parkinson’s disease. BMB Rep..

[166] Khan M.T., Irfan M., Ahsan H., Ahmed A., Kaushik A.C., Khan A.S., Chinnasamy S., Ali A., Wei D.Q. (2021). Structures of SARS-CoV-2 RNA-Binding Proteins and Therapeutic Targets. Intervirology.

[167] Semerdzhiev S.A., Fakhree M.A.A., Segers-Nolten I., Blum C., Claessens M. (2022). Interactions between SARS-CoV-2 N-Protein and α-Synuclein Accelerate Amyloid Formation. ACS Chem. Neurosci..

[168] Malkus K.A., Tsika E., Ischiropoulos H. (2009). Oxidative modifications, mitochondrial dysfunction, and impaired protein degradation in Parkinson’s disease: How neurons are lost in the Bermuda triangle. Mol. Neurodegener..

[169] Zeng X.S., Geng W.S., Jia J.J., Chen L., Zhang P.P. (2018). Cellular and Molecular Basis of Neurodegeneration in Parkinson Disease. Front. Aging Neurosci..

[170] Fujita K.A., Ostaszewski M., Matsuoka Y., Ghosh S., Glaab E., Trefois C., Crespo I., Perumal T.M., Jurkowski W., Antony P.M. (2014). Integrating pathways of Parkinson’s disease in a molecular interaction map. Mol. Neurobiol..

[171] Henchcliffe C., Beal M.F. (2008). Mitochondrial biology and oxidative stress in Parkinson disease pathogenesis. Nat. Clin. Pract. Neurol..

[172] Abou-Sleiman P.M., Muqit M.M., Wood N.W. (2006). Expanding insights of mitochondrial dysfunction in Parkinson’ disease. Nat. Rev. Neurosci..

[173] Brieger K., Schiavone S., Miller F.J., Krause K.H. (2012). Reactive oxygen species: From health to disease. Swiss Med. Wkly..

[174] Kannan K., Jain S.K. (2000). Oxidative stress and apoptosis. Pathophysiol. Off. J. Int. Soc. Pathophysiol..

[175] Subramaniam S.R., Vergnes L., Franich N.R., Reue K., Chesselet M.F. (2014). Region specific mitochondrial impairment in mice with widespread overexpression of alpha-synuclein. Neurobiol. Dis..

[176] Hattori N., Tanaka M., Ozawa T., Mizuno Y. (1991). Immunohistochemical studies on complexes I, II, III, and IV of mitochondria in Parkinson’s disease. Ann. Neurol..

[177] Parker W.D., Parks J.K., Swerdlow R.H. (2008). Complex I deficiency in Parkinson’s disease frontal cortex. Brain Res..

[178] Martin L.J., Pan Y., Price A.C., Sterling W., Copeland N.G., Jenkins N.A., Price D.L., Lee M.K. (2006). Parkinson’s disease alpha-synuclein transgenic mice develop neuronal mitochondrial degeneration and cell death. J. Neurosci. Off. J. Soc. Neurosci..

[179] Gatti P., Ilamathi H.S., Todkar K., Germain M. (2020). Mitochondria Targeted Viral Replication and Survival Strategies-Prospective on SARS-CoV-2. Front. Pharmacol..

[180] Singh K.K., Chaubey G., Chen J.Y., Suravajhala P. (2020). Decoding SARS-CoV-2 hijacking of host mitochondria in COVID-19 pathogenesis. Am. J. Physiol. Cell Physiol..

[181] Burtscher J., Cappellano G., Omori A., Koshiba T., Millet G.P. (2020). Mitochondria: In the Cross Fire of SARS-CoV-2 and Immunity. iScience.

[182] Morowitz J.M., Pogson K.B., Roque D.A., Church F.C. (2022). Role of SARS-CoV-2 in Modifying Neurodegenerative Processes in Parkinson’s Disease: A Narrative Review. Brain Sci..

[183] Suhail S., Zajac J., Fossum C., Lowater H., McCracken C., Severson N., Laatsch B., Narkiewicz-Jodko A., Johnson B., Liebau J. (2020). Role of Oxidative Stress on SARS-CoV (SARS) and SARS-CoV-2 (COVID-19) Infection: A Review. Protein J..

[184] Chernyak B.V., Popova E.N., Prikhodko A.S., Grebenchikov O.A., Zinovkina L.A., Zinovkin R.A. (2020). COVID-19 and Oxidative Stress. Biochem. Biokhimiia.

[185] Shang C., Liu Z., Zhu Y., Lu J., Ge C., Zhang C., Li N., Jin N., Li Y., Tian M. (2021). SARS-CoV-2 Causes Mitochondrial Dysfunction and Mitophagy Impairment. Front. Microbiol..

[186] Tiku V., Tan M.W., Dikic I. (2020). Mitochondrial Functions in Infection and Immunity. Trends Cell Biol..

[187] Shi T.T., Yang F.Y., Liu C., Cao X., Lu J., Zhang X.L., Yuan M.X., Chen C., Yang J.K. (2018). Angiotensin-converting enzyme 2 regulates mitochondrial function in pancreatic β-cells. Biochem. Biophys. Res. Commun..

[188] Bordt E.A., Polster B.M. (2014). NADPH oxidase- and mitochondria-derived reactive oxygen species in proinflammatory microglial activation: A bipartisan affair?. Free Radic. Biol. Med..

[189] Clough E., Inigo J., Chandra D., Chaves L., Reynolds J.L., Aalinkeel R., Schwartz S.A., Khmaladze A., Mahajan S.D. (2021). Mitochondrial Dynamics in SARS-COV2 Spike Protein Treated Human Microglia: Implications for Neuro-COVID. J. Neuroimmune Pharmacol. Off. J. Soc. NeuroImmune Pharmacol..

[190] Omura T., Kaneko M., Okuma Y., Matsubara K., Nomura Y. (2013). Endoplasmic reticulum stress and Parkinson’s disease: The role of HRD1 in averting apoptosis in neurodegenerative disease. Oxidative Med. Cell. Longev..

[191] Colla E. (2019). Linking the Endoplasmic Reticulum to Parkinson’s Disease and Alpha-Synucleinopathy. Front. Neurosci..

[192] Hitomi J., Katayama T., Eguchi Y., Kudo T., Taniguchi M., Koyama Y., Manabe T., Yamagishi S., Bando Y., Imaizumi K. (2004). Involvement of caspase-4 in endoplasmic reticulum stress-induced apoptosis and Abeta-induced cell death. J. Cell Biol..

[193] Marciniak S.J., Yun C.Y., Oyadomari S., Novoa I., Zhang Y., Jungreis R., Nagata K., Harding H.P., Ron D. (2004). CHOP induces death by promoting protein synthesis and oxidation in the stressed endoplasmic reticulum. Genes Dev..

[194] Bartolini D., Stabile A.M., Vacca C., Pistilli A., Rende M., Gioiello A., Cruciani G., Galli F. (2022). Endoplasmic reticulum stress and NF-kB activation in SARS-CoV-2 infected cells and their response to antiviral therapy. IUBMB Life.

[195] Gordon D.E., Jang G.M., Bouhaddou M., Xu J., Obernier K., White K.M., O’Meara M.J., Rezelj V.V., Guo J.Z., Swaney D.L. (2020). A SARS-CoV-2 protein interaction map reveals targets for drug repurposing. Nature.

[196] Rashid F., Dzakah E.E., Wang H., Tang S. (2021). The ORF8 protein of SARS-CoV-2 induced endoplasmic reticulum stress and mediated immune evasion by antagonizing production of interferon beta. Virus Res..

[197] Jiang P., Gan M., Ebrahim A.S., Lin W.L., Melrose H.L., Yen S.H. (2010). ER stress response plays an important role in aggregation of α-synuclein. Mol. Neurodegener..

[198] Chaudhari N., Talwar P., Parimisetty A., Lefebvre d’Hellencourt C., Ravanan P. (2014). A molecular web: Endoplasmic reticulum stress, inflammation, and oxidative stress. Front. Cell. Neurosci..

[199] Jones J.T., Qian X., van der Velden J.L., Chia S.B., McMillan D.H., Flemer S., Hoffman S.M., Lahue K.G., Schneider R.W., Nolin J.D. (2016). Glutathione S-transferase pi modulates NF-κB activation and pro-inflammatory responses in lung epithelial cells. Redox Biol..

[200] Li W., Qiao J., You Q., Zong S., Peng Q., Liu Y., Hu S., Liu W., Li S., Shu X. (2021). SARS-CoV-2 Nsp5 Activates NF-κB Pathway by Upregulating SUMOylation of MAVS. Front. Immunol..

[201] Hunot S., Brugg B., Ricard D., Michel P.P., Muriel M.P., Ruberg M., Faucheux B.A., Agid Y., Hirsch E.C. (1997). Nuclear translocation of NF-kappaB is increased in dopaminergic neurons of patients with parkinson disease. Proc. Natl. Acad. Sci. USA.

[202] Dolatshahi M., Ranjbar Hameghavandi M.H., Sabahi M., Rostamkhani S. (2021). Nuclear factor-kappa B (NF-κB) in pathophysiology of Parkinson disease: Diverse patterns and mechanisms contributing to neurodegeneration. Eur. J. Neurosci..

[203] Bellucci A., Bubacco L., Longhena F., Parrella E., Faustini G., Porrini V., Bono F., Missale C., Pizzi M. (2020). Nuclear Factor-κB Dysregulation and α-Synuclein Pathology: Critical Interplay in the Pathogenesis of Parkinson’s Disease. Front. Aging Neurosci..

[204] Singh S.S., Rai S.N., Birla H., Zahra W., Rathore A.S., Singh S.P. (2020). NF-κB-Mediated Neuroinflammation in Parkinson’s Disease and Potential Therapeutic Effect of Polyphenols. Neurotox. Res..

[205] Ebrahimi-Fakhari D., Cantuti-Castelvetri I., Fan Z., Rockenstein E., Masliah E., Hyman B.T., McLean P.J., Unni V.K. (2011). Distinct roles in vivo for the ubiquitin-proteasome system and the autophagy-lysosomal pathway in the degradation of α-synuclein. J. Neurosci. Off. J. Soc. Neurosci..

[206] Dehay B., Martinez-Vicente M., Caldwell G.A., Caldwell K.A., Yue Z., Cookson M.R., Klein C., Vila M., Bezard E. (2013). Lysosomal impairment in Parkinson’s disease. Mov. Disord. Off. J. Mov. Disord. Soc..

[207] Lakshmana M.K. (2022). SARS-CoV-2-induced autophagy dysregulation may cause neuronal dysfunction in COVID-19. Neural Regen. Res..

[208] Singh K., Chen Y.C., Hassanzadeh S., Han K., Judy J.T., Seifuddin F., Tunc I., Sack M.N., Pirooznia M. (2021). Network Analysis and Transcriptome Profiling Identify Autophagic and Mitochondrial Dysfunctions in SARS-CoV-2 Infection. Front. Genet..

[209] Miao G., Zhao H., Li Y., Ji M., Chen Y., Shi Y., Bi Y., Wang P., Zhang H. (2021). ORF3a of the COVID-19 virus SARS-CoV-2 blocks HOPS complex-mediated assembly of the SNARE complex required for autolysosome formation. Dev. Cell.

[210] Hou P., Wang X., Wang H., Wang T., Yu Z., Xu C., Zhao Y., Wang W., Zhao Y., Chu F. (2022). The ORF7a protein of SARS-CoV-2 initiates autophagy and limits autophagosome-lysosome fusion via degradation of SNAP29 to promote virus replication. Autophagy.

[211] Mohamud Y., Xue Y.C., Liu H., Ng C.S., Bahreyni A., Jan E., Luo H. (2021). The papain-like protease of coronaviruses cleaves ULK1 to disrupt host autophagy. Biochem. Biophys. Res. Commun..

[212] Russell R.C., Tian Y., Yuan H., Park H.W., Chang Y.Y., Kim J., Kim H., Neufeld T.P., Dillin A., Guan K.L. (2013). ULK1 induces autophagy by phosphorylating Beclin-1 and activating VPS34 lipid kinase. Nat. Cell Biol..

[213] García-Pérez B.E., González-Rojas J.A., Salazar M.I., Torres-Torres C., Castrejón-Jiménez N.S. (2020). Taming the Autophagy as a Strategy for Treating COVID-19. Cells.

[214] Zaborowski M.P., Balaj L., Breakefield X.O., Lai C.P. (2015). Extracellular Vesicles: Composition, Biological Relevance, and Methods of Study. Bioscience.

[215] Caby M.P., Lankar D., Vincendeau-Scherrer C., Raposo G., Bonnerot C. (2005). Exosomal-like vesicles are present in human blood plasma. Int. Immunol..

[216] Zlotogorski-Hurvitz A., Dayan D., Chaushu G., Korvala J., Salo T., Sormunen R., Vered M. (2015). Human saliva-derived exosomes: Comparing methods of isolation. J. Histochem. Cytochem. Off. J. Histochem. Soc..

[217] Simpson R.J., Lim J.W., Moritz R.L., Mathivanan S. (2009). Exosomes: Proteomic insights and diagnostic potential. Expert Rev. Proteom..

[218] Valadi H., Ekström K., Bossios A., Sjöstrand M., Lee J.J., Lötvall J.O. (2007). Exosome-mediated transfer of mRNAs and microRNAs is a novel mechanism of genetic exchange between cells. Nat. Cell Biol..

[219] Yáñez-Mó M., Siljander P.R., Andreu Z., Zavec A.B., Borràs F.E., Buzas E.I., Buzas K., Casal E., Cappello F., Carvalho J. (2015). Biological properties of extracellular vesicles and their physiological functions. J. Extracell. Vesicles.

[220] Jung M.K., Mun J.Y. (2018). Sample Preparation and Imaging of Exosomes by Transmission Electron Microscopy. J. Vis. Exp. JoVE.

[221] Borges F.T., Reis L.A., Schor N. (2013). Extracellular vesicles: Structure, function, and potential clinical uses in renal diseases. Braz. J. Med. Biol. Res. Rev. Bras. Pesqui. Med. E Biol..

[222] Bebelman M.P., Smit M.J., Pegtel D.M., Baglio S.R. (2018). Biogenesis and function of extracellular vesicles in cancer. Pharmacol. Ther..

[223] Doyle L.M., Wang M.Z. (2019). Overview of Extracellular Vesicles, Their Origin, Composition, Purpose, and Methods for Exosome Isolation and Analysis. Cells.

[224] Kalluri R., LeBleu V.S. (2020). The biology, function, and biomedical applications of exosomes. Science.

[225] Hoffman H.K., Fernandez M.V., Groves N.S., Freed E.O., van Engelenburg S.B. (2019). Genomic tagging of endogenous human ESCRT-I complex preserves ESCRT-mediated membrane-remodeling functions. J. Biol. Chem..

[226] van Niel G., Porto-Carreiro I., Simoes S., Raposo G. (2006). Exosomes: A common pathway for a specialized function. J. Biochem..

[227] Gurung S., Perocheau D., Touramanidou L., Baruteau J. (2021). The exosome journey: From biogenesis to uptake and intracellular signalling. Cell Commun. Signal. CCS.

[228] Stuffers S., Sem Wegner C., Stenmark H., Brech A. (2009). Multivesicular endosome biogenesis in the absence of ESCRTs. Traffic.

[229] Hoshino A., Costa-Silva B., Shen T.L., Rodrigues G., Hashimoto A., Tesic Mark M., Molina H., Kohsaka S., Di Giannatale A., Ceder S. (2015). Tumour exosome integrins determine organotropic metastasis. Nature.

[230] Miyanishi M., Tada K., Koike M., Uchiyama Y., Kitamura T., Nagata S. (2007). Identification of Tim4 as a phosphatidylserine receptor. Nature.

[231] Mathieu M., Martin-Jaular L., Lavieu G., Théry C. (2019). Specificities of secretion and uptake of exosomes and other extracellular vesicles for cell-to-cell communication. Nat. Cell Biol..

[232] Larssen P., Wik L., Czarnewski P., Eldh M., Löf L., Ronquist K.G., Dubois L., Freyhult E., Gallant C.J., Oelrich J. (2017). Tracing Cellular Origin of Human Exosomes Using Multiplex Proximity Extension Assays. Mol. Cell. Proteom. MCP.

[233] Anastasi F., Masciandaro S.M., Carratore R.D., Dell’Anno M.T., Signore G., Falleni A., McDonnell L.A., Bongioanni P. (2021). Proteomics Profiling of Neuron-Derived Small Extracellular Vesicles from Human Plasma: Enabling Single-Subject Analysis. Int. J. Mol. Sci..

[234] Banks W.A., Sharma P., Bullock K.M., Hansen K.M., Ludwig N., Whiteside T.L. (2020). Transport of Extracellular Vesicles across the Blood-Brain Barrier: Brain Pharmacokinetics and Effects of Inflammation. Int. J. Mol. Sci..

[235] Hornung S., Dutta S., Bitan G. (2020). CNS-Derived Blood Exosomes as a Promising Source of Biomarkers: Opportunities and Challenges. Front. Mol. Neurosci..

[236] Liu W., Bai X., Zhang A., Huang J., Xu S., Zhang J. (2019). Role of Exosomes in Central Nervous System Diseases. Front. Mol. Neurosci..

[237] Alvarez-Erviti L., Seow Y., Schapira A.H., Gardiner C., Sargent I.L., Wood M.J., Cooper J.M. (2011). Lysosomal dysfunction increases exosome-mediated alpha-synuclein release and transmission. Neurobiol. Dis..

[238] Shi M., Liu C., Cook T.J., Bullock K.M., Zhao Y., Ginghina C., Li Y., Aro P., Dator R., He C. (2014). Plasma exosomal α-synuclein is likely CNS-derived and increased in Parkinson’s disease. Acta Neuropathol..

[239] Si X., Tian J., Chen Y., Yan Y., Pu J., Zhang B. (2019). Central Nervous System-Derived Exosomal Alpha-Synuclein in Serum May Be a Biomarker in Parkinson’s Disease. Neuroscience.

[240] Olanow C.W., Brundin P. (2013). Parkinson’s disease and alpha synuclein: Is Parkinson’s disease a prion-like disorder?. Mov. Disord. Off. J. Mov. Disord. Soc..

[241] Peng C., Trojanowski J.Q., Lee V.M. (2020). Protein transmission in neurodegenerative disease. Nat. Rev. Neurol..

[242] Danzer K.M., Kranich L.R., Ruf W.P., Cagsal-Getkin O., Winslow A.R., Zhu L., Vanderburg C.R., McLean P.J. (2012). Exosomal cell-to-cell transmission of alpha synuclein oligomers. Mol. Neurodegener..

[243] Fan R.Z., Guo M., Luo S., Cui M., Tieu K. (2019). Exosome release and neuropathology induced by α-synuclein: New insights into protective mechanisms of Drp1 inhibition. Acta Neuropathol. Commun..

[244] Desplats P., Lee H.J., Bae E.J., Patrick C., Rockenstein E., Crews L., Spencer B., Masliah E., Lee S.J. (2009). Inclusion formation and neuronal cell death through neuron-to-neuron transmission of alpha-synuclein. Proc. Natl. Acad. Sci. USA.

[245] Chang C., Lang H., Geng N., Wang J., Li N., Wang X. (2013). Exosomes of BV-2 cells induced by alpha-synuclein: Important mediator of neurodegeneration in PD. Neurosci. Lett..

[246] Stuendl A., Kunadt M., Kruse N., Bartels C., Moebius W., Danzer K.M., Mollenhauer B., Schneider A. (2016). Induction of α-synuclein aggregate formation by CSF exosomes from patients with Parkinson’s disease and dementia with Lewy bodies. Brain J. Neurol..

[247] Han C., Xiong N., Guo X., Huang J., Ma K., Liu L., Xia Y., Shen Y., Li J., Jiang H. (2019). Exosomes from patients with Parkinson’s disease are pathological in mice. J. Mol. Med..

[248] Si X.L., Fang Y.J., Li L.F., Gu L.Y., Yin X.Z., Jun T., Yan Y.P., Pu J.L., Zhang B.R. (2021). From inflammasome to Parkinson’s disease: Does the NLRP3 inflammasome facilitate exosome secretion and exosomal alpha-synuclein transmission in Parkinson’s disease?. Exp. Neurol..

[249] Vandendriessche C., Bruggeman A., Van Cauwenberghe C., Vandenbroucke R.E. (2020). Extracellular Vesicles in Alzheimer’s and Parkinson’s Disease: Small Entities with Large Consequences. Cells.

[250] Li K.L., Huang H.Y., Ren H., Yang X.L. (2022). Role of exosomes in the pathogenesis of inflammation in Parkinson’s disease. Neural Regen. Res..

[251] Gao H.M., Hong J.S. (2008). Why neurodegenerative diseases are progressive: Uncontrolled inflammation drives disease progression. Trends Immunol..

[252] Brück D., Wenning G.K., Stefanova N., Fellner L. (2016). Glia and alpha-synuclein in neurodegeneration: A complex interaction. Neurobiol. Dis..

[253] Rocha E.M., De Miranda B., Sanders L.H. (2018). Alpha-synuclein: Pathology, mitochondrial dysfunction and neuroinflammation in Parkinson’s disease. Neurobiol. Dis..

[254] Xia Y., Zhang G., Kou L., Yin S., Han C., Hu J., Wan F., Sun Y., Wu J., Li Y. (2021). Reactive microglia enhance the transmission of exosomal α-synuclein via toll-like receptor 2. Brain J. Neurol..

[255] Guo M., Wang J., Zhao Y., Feng Y., Han S., Dong Q., Cui M., Tieu K. (2020). Microglial exosomes facilitate α-synuclein transmission in Parkinson’s disease. Brain J. Neurol..

[256] Sepúlveda D., Cisternas-Olmedo M., Arcos J., Nassif M., Vidal R.L. (2022). Contribution of Autophagy-Lysosomal Pathway in the Exosomal Secretion of Alpha-Synuclein and Its Impact in the Progression of Parkinson’s Disease. Front. Mol. Neurosci..

[257] Rattanavirotkul N., Kirschner K., Chandra T. (2021). Induction and transmission of oncogene-induced senescence. Cell. Mol. Life Sci. CMLS.

[258] Borghesan M., Fafián-Labora J., Eleftheriadou O., Carpintero-Fernández P., Paez-Ribes M., Vizcay-Barrena G., Swisa A., Kolodkin-Gal D., Ximénez-Embún P., Lowe R. (2019). Small Extracellular Vesicles Are Key Regulators of Non-cell Autonomous Intercellular Communication in Senescence via the Interferon Protein IFITM3. Cell Rep..

[259] Chinta S.J., Woods G., Demaria M., Rane A., Zou Y., McQuade A., Rajagopalan S., Limbad C., Madden D.T., Campisi J. (2018). Cellular Senescence Is Induced by the Environmental Neurotoxin Paraquat and Contributes to Neuropathology Linked to Parkinson’s Disease. Cell Rep..

[260] Fafián-Labora J.A., Rodríguez-Navarro J.A., O’Loghlen A. (2020). Small Extracellular Vesicles Have GST Activity and Ameliorate Senescence-Related Tissue Damage. Cell Metab..

[261] Terlecki-Zaniewicz L., Lämmermann I., Latreille J., Bobbili M.R., Pils V., Schosserer M., Weinmüllner R., Dellago H., Skalicky S., Pum D. (2018). Small extracellular vesicles and their miRNA cargo are anti-apoptotic members of the senescence-associated secretory phenotype. Aging.

[262] Jankovic J., Rajput A.H., McDermott M.P., Perl D.P. (2000). The evolution of diagnosis in early Parkinson disease. Parkinson Study Group. Arch. Neurol..

[263] Adler C.H., Beach T.G., Hentz J.G., Shill H.A., Caviness J.N., Driver-Dunckley E., Sabbagh M.N., Sue L.I., Jacobson S.A., Belden C.M. (2014). Low clinical diagnostic accuracy of early vs advanced Parkinson disease: Clinicopathologic study. Neurology.

[264] Niu M., Li Y., Li G., Zhou L., Luo N., Yao M., Kang W., Liu J. (2020). A longitudinal study on α-synuclein in plasma neuronal exosomes as a biomarker for Parkinson’s disease development and progression. Eur. J. Neurol..

[265] Cao Z., Wu Y., Liu G., Jiang Y., Wang X., Wang Z., Feng T. (2019). α-Synuclein in salivary extracellular vesicles as a potential biomarker of Parkinson’s disease. Neurosci. Lett..

[266] Jiang C., Hopfner F., Katsikoudi A., Hein R., Catli C., Evetts S., Huang Y., Wang H., Ryder J.W., Kuhlenbaeumer G. (2020). Serum neuronal exosomes predict and differentiate Parkinson’s disease from atypical parkinsonism. J. Neurol. Neurosurg. Psychiatry.

[267] Shi M., Kovac A., Korff A., Cook T.J., Ginghina C., Bullock K.M., Yang L., Stewart T., Zheng D., Aro P. (2016). CNS tau efflux via exosomes is likely increased in Parkinson’s disease but not in Alzheimer’s disease. Alzheimer’s Dement. J. Alzheimer’s Assoc..

[268] Kitamura Y., Kojima M., Kurosawa T., Sasaki R., Ichihara S., Hiraku Y., Tomimoto H., Murata M., Oikawa S. (2018). Proteomic Profiling of Exosomal Proteins for Blood-based Biomarkers in Parkinson’s Disease. Neuroscience.

[269] Zhao Z.H., Chen Z.T., Zhou R.L., Zhang X., Ye Q.Y., Wang Y.Z. (2018). Increased DJ-1 and α-Synuclein in Plasma Neural-Derived Exosomes as Potential Markers for Parkinson’s Disease. Front. Aging Neurosci..

[270] Xia X., Wang Y., Huang Y., Zhang H., Lu H., Zheng J.C. (2019). Exosomal miRNAs in central nervous system diseases: Biomarkers, pathological mediators, protective factors and therapeutic agents. Prog. Neurobiol..

[271] Maciotta S., Meregalli M., Torrente Y. (2013). The involvement of microRNAs in neurodegenerative diseases. Front. Cell. Neurosci..

[272] Li D., Li Y.P., Li Y.X., Zhu X.H., Du X.G., Zhou M., Li W.B., Deng H.Y. (2018). Effect of Regulatory Network of Exosomes and microRNAs on Neurodegenerative Diseases. Chin. Med. J..

[273] Paschon V., Takada S.H., Ikebara J.M., Sousa E., Raeisossadati R., Ulrich H., Kihara A.H. (2016). Interplay Between Exosomes, microRNAs and Toll-Like Receptors in Brain Disorders. Mol. Neurobiol..

[274] Wang X., Zhou Y., Gao Q., Ping D., Wang Y., Wu W., Lin X., Fang Y., Zhang J., Shao A. (2020). The Role of Exosomal microRNAs and Oxidative Stress in Neurodegenerative Diseases. Oxid. Med. Cell. Longev..

[275] Gui Y., Liu H., Zhang L., Lv W., Hu X. (2015). Altered microRNA profiles in cerebrospinal fluid exosome in Parkinson disease and Alzheimer disease. Oncotarget.

[276] Yao Y.F., Qu M.W., Li G.C., Zhang F.B., Rui H.C. (2018). Circulating exosomal miRNAs as diagnostic biomarkers in Parkinson’s disease. Eur. Rev. Med. Pharmacol. Sci..

[277] He S., Huang L., Shao C., Nie T., Xia L., Cui B., Lu F., Zhu L., Chen B., Yang Q. (2021). Several miRNAs derived from serum extracellular vesicles are potential biomarkers for early diagnosis and progression of Parkinson’s disease. Transl. Neurodegener..

[278] Chaudhari P., Ghate V., Nampoothiri M., Lewis S. (2022). Multifunctional role of exosomes in viral diseases: From transmission to diagnosis and therapy. Cell. Signal..

[279] van Dongen H.M., Masoumi N., Witwer K.W., Pegtel D.M. (2016). Extracellular Vesicles Exploit Viral Entry Routes for Cargo Delivery. Microbiol. Mol. Biol. Rev. MMBR.

[280] Nolte-‘t Hoen E., Cremer T., Gallo R.C., Margolis L.B. (2016). Extracellular vesicles and viruses: Are they close relatives?. Proc. Natl. Acad. Sci. USA.

[281] Gunasekaran M., Bansal S., Ravichandran R., Sharma M., Perincheri S., Rodriguez F., Hachem R., Fisher C.E., Limaye A.P., Omar A. (2020). Respiratory viral infection in lung transplantation induces exosomes that trigger chronic rejection. J. Heart Lung Transplant. Off. Publ. Int. Soc. Heart Transplant..

[282] Kuate S., Cinatl J., Doerr H.W., Uberla K. (2007). Exosomal vaccines containing the S protein of the SARS coronavirus induce high levels of neutralizing antibodies. Virology.

[283] Peluso M.J., Deeks S.G., Mustapic M., Kapogiannis D., Henrich T.J., Lu S., Goldberg S.A., Hoh R., Chen J.Y., Martinez E.O. (2022). SARS-CoV-2 and Mitochondrial Proteins in Neural-Derived Exosomes of COVID-19. Ann. Neurol..

[284] Pesce E., Manfrini N., Cordiglieri C., Santi S., Bandera A., Gobbini A., Gruarin P., Favalli A., Bombaci M., Cuomo A. (2021). Exosomes Recovered From the Plasma of COVID-19 Patients Expose SARS-CoV-2 Spike-Derived Fragments and Contribute to the Adaptive Immune Response. Front. Immunol..

[285] Barberis E., Vanella V.V., Falasca M., Caneapero V., Cappellano G., Raineri D., Ghirimoldi M., De Giorgis V., Puricelli C., Vaschetto R. (2021). Circulating Exosomes Are Strongly Involved in SARS-CoV-2 Infection. Front. Mol. Biosci..

[286] Gould S.J., Booth A.M., Hildreth J.E. (2003). The Trojan exosome hypothesis. Proc. Natl. Acad. Sci. USA.

[287] Jackson C.B., Farzan M., Chen B., Choe H. (2022). Mechanisms of SARS-CoV-2 entry into cells. Nat. Rev. Mol. Cell Biol..

[288] Wang J., Chen S., Bihl J. (2020). Exosome-Mediated Transfer of ACE2 (Angiotensin-Converting Enzyme 2) from Endothelial Progenitor Cells Promotes Survival and Function of Endothelial Cell. Oxidative Med. Cell. Longev..

[289] Inal J.M. (2020). Decoy ACE2-expressing extracellular vesicles that competitively bind SARS-CoV-2 as a possible COVID-19 therapy. Clin. Sci..

[290] Schorey J.S., Cheng Y., Singh P.P., Smith V.L. (2015). Exosomes and other extracellular vesicles in host-pathogen interactions. EMBO Rep..

[291] Böker K.O., Lemus-Diaz N., Rinaldi Ferreira R., Schiller L., Schneider S., Gruber J. (2018). The Impact of the CD9 Tetraspanin on Lentivirus Infectivity and Exosome Secretion. Mol. Ther. J. Am. Soc. Gene Ther..

[292] Earnest J.T., Hantak M.P., Li K., McCray P.B., Perlman S., Gallagher T. (2017). The tetraspanin CD9 facilitates MERS-coronavirus entry by scaffolding host cell receptors and proteases. PLoS Pathog..

[293] Lam S.M., Zhang C., Wang Z., Ni Z., Zhang S., Yang S., Huang X., Mo L., Li J., Lee B. (2021). A multi-omics investigation of the composition and function of extracellular vesicles along the temporal trajectory of COVID-19. Nat. Metab..

[294] Wu X., Zheng T., Zhang B. (2017). Exosomes in Parkinson’s Disease. Neurosci. Bull..

[295] Horn M.D., MacLean A.G. (2021). Extracellular Vesicles as a Means of Viral Immune Evasion, CNS Invasion, and Glia-Induced Neurodegeneration. Front. Cell. Neurosci..

[296] Miller A.A., Spencer S.J. (2014). Obesity and neuroinflammation: A pathway to cognitive impairment. Brain Behav. Immun..

[297] Fuggle N.R., Howe F.A., Allen R.L., Sofat N. (2014). New insights into the impact of neuro-inflammation in rheumatoid arthritis. Front. Neurosci..

[298] Henry C.J., Huang Y., Wynne A.M., Godbout J.P. (2009). Peripheral lipopolysaccharide (LPS) challenge promotes microglial hyperactivity in aged mice that is associated with exaggerated induction of both pro-inflammatory IL-1beta and anti-inflammatory IL-10 cytokines. Brain Behav. Immun..

[299] Posey K.A., Clegg D.J., Printz R.L., Byun J., Morton G.J., Vivekanandan-Giri A., Pennathur S., Baskin D.G., Heinecke J.W., Woods S.C. (2009). Hypothalamic proinflammatory lipid accumulation, inflammation, and insulin resistance in rats fed a high-fat diet. Am. J. Physiol. Endocrinol. Metab..

[300] Li J.J., Wang B., Kodali M.C., Chen C., Kim E., Patters B.J., Lan L., Kumar S., Wang X., Yue J. (2018). In vivo evidence for the contribution of peripheral circulating inflammatory exosomes to neuroinflammation. J. Neuroinflamm..

[301] Gupta A., Pulliam L. (2014). Exosomes as mediators of neuroinflammation. J. Neuroinflamm..

[302] Bianco F., Pravettoni E., Colombo A., Schenk U., Möller T., Matteoli M., Verderio C. (2005). Astrocyte-derived ATP induces vesicle shedding and IL-1 beta release from microglia. J. Immunol..

[303] Bliederhaeuser C., Grozdanov V., Speidel A., Zondler L., Ruf W.P., Bayer H., Kiechle M., Feiler M.S., Freischmidt A., Brenner D. (2016). Age-dependent defects of alpha-synuclein oligomer uptake in microglia and monocytes. Acta Neuropathol..

[304] Sur S., Khatun M., Steele R., Isbell T.S., Ray R., Ray R.B. (2021). Exosomes from COVID-19 Patients Carry Tenascin-C and Fibrinogen-β in Triggering Inflammatory Signals in Cells of Distant Organ. Int. J. Mol. Sci..

[305] Hofer M.J., Li W., Lim S.L., Campbell I.L. (2010). The type I interferon-alpha mediates a more severe neurological disease in the absence of the canonical signaling molecule interferon regulatory factor 9. J. Neurosci. Off. J. Soc. Neurosci..

[306] Ottum P.A., Arellano G., Reyes L.I., Iruretagoyena M., Naves R. (2015). Opposing Roles of Interferon-Gamma on Cells of the Central Nervous System in Autoimmune Neuroinflammation. Front. Immunol..

[307] Mishra R., Banerjea A.C. (2021). SARS-CoV-2 Spike Targets USP33-IRF9 Axis via Exosomal miR-148a to Activate Human Microglia. Front. Immunol..

[308] Williams G.P., Schonhoff A.M., Jurkuvenaite A., Gallups N.J., Standaert D.G., Harms A.S. (2021). CD4 T cells mediate brain inflammation and neurodegeneration in a mouse model of Parkinson’s disease. Brain J. Neurol..

[309] Brochard V., Combadière B., Prigent A., Laouar Y., Perrin A., Beray-Berthat V., Bonduelle O., Alvarez-Fischer D., Callebert J., Launay J.M. (2009). Infiltration of CD4+ lymphocytes into the brain contributes to neurodegeneration in a mouse model of Parkinson disease. J. Clin. Investig..

[310] Fang P., Schachner M., Shen Y.Q. (2012). HMGB1 in development and diseases of the central nervous system. Mol. Neurobiol..

[311] Santoro M., Maetzler W., Stathakos P., Martin H.L., Hobert M.A., Rattay T.W., Gasser T., Forrester J.V., Berg D., Tracey K.J. (2016). In-vivo evidence that high mobility group box 1 exerts deleterious effects in the 1-methyl-4-phenyl-1,2,3,6-tetrahydropyridine model and Parkinson’s disease which can be attenuated by glycyrrhizin. Neurobiol. Dis..

[312] Sasaki T., Liu K., Agari T., Yasuhara T., Morimoto J., Okazaki M., Takeuchi H., Toyoshima A., Sasada S., Shinko A. (2016). Anti-high mobility group box 1 antibody exerts neuroprotection in a rat model of Parkinson’s disease. Exp. Neurol..

[313] Canaslan S., Schmitz M., Villar-Piqué A., Maass F., Gmitterová K., Varges D., Lingor P., Llorens F., Hermann P., Zerr I. (2021). Detection of Cerebrospinal Fluid Neurofilament Light Chain as a Marker for Alpha-Synucleinopathies. Front. Aging Neurosci..

[314] Bäckström D., Linder J., Jakobson Mo S., Riklund K., Zetterberg H., Blennow K., Forsgren L., Lenfeldt N. (2020). NfL as a biomarker for neurodegeneration and survival in Parkinson disease. Neurology.

[315] Srpova B., Uher T., Hrnciarova T., Barro C., Andelova M., Michalak Z., Vaneckova M., Krasensky J., Noskova L., Havrdova E.K. (2021). Serum neurofilament light chain reflects inflammation-driven neurodegeneration and predicts delayed brain volume loss in early stage of multiple sclerosis. Mult. Scler..

[316] Sun B., Tang N., Peluso M.J., Iyer N.S., Torres L., Donatelli J.L., Munter S.E., Nixon C.C., Rutishauser R.L., Rodriguez-Barraquer I. (2021). Characterization and Biomarker Analyses of Post-COVID-19 Complications and Neurological Manifestations. Cells.

[317] Butowt R., von Bartheld C.S. (2022). The route of SARS-CoV-2 to brain infection: Have we been barking up the wrong tree?. Mol. Neurodegener..

[318] Bulfamante G., Bocci T., Falleni M., Campiglio L., Coppola S., Tosi D., Chiumello D., Priori A. (2021). Brainstem neuropathology in two cases of COVID-19: SARS-CoV-2 trafficking between brain and lung. J. Neurol..

[319] Rangon C.M., Krantic S., Moyse E., Fougère B. (2020). The Vagal Autonomic Pathway of COVID-19 at the Crossroad of Alzheimer’s Disease and Aging: A Review of Knowledge. J. Alzheimer’s Dis. Rep..

[320] Fenrich M., Mrdenovic S., Balog M., Tomic S., Zjalic M., Roncevic A., Mandic D., Debeljak Z., Heffer M. (2020). SARS-CoV-2 Dissemination Through Peripheral Nerves Explains Multiple Organ Injury. Front. Cell. Neurosci..

[321] Sarubbo F., El Haji K., Vidal-Balle A., Bargay Lleonart J. (2022). Neurological consequences of COVID-19 and brain related pathogenic mechanisms: A new challenge for neuroscience. Brain Behav. Immun.-Health.

[322] Höglinger G.U., Alvarez-Fischer D., Arias-Carrión O., Djufri M., Windolph A., Keber U., Borta A., Ries V., Schwarting R.K., Scheller D. (2015). A new dopaminergic nigro-olfactory projection. Acta Neuropathol..

[323] Otake K., Oiso Y., Mitsuma T., Hirooka Y., Adachi K. (1994). Hypothalamic dysfunction in Parkinson’s disease patients. Acta Med. Hung..

[324] Greene J.G. (2014). Causes and consequences of degeneration of the dorsal motor nucleus of the vagus nerve in Parkinson’s disease. Antioxid. Redox Signal..

[325] Thakur K.T., Miller E.H., Glendinning M.D., Al-Dalahmah O., Banu M.A., Boehme A.K., Boubour A.L., Bruce S.S., Chong A.M., Claassen J. (2021). COVID-19 neuropathology at Columbia University Irving Medical Center/New York Presbyterian Hospital. Brain J. Neurol..

[326] Käufer C., Schreiber C.S., Hartke A.S., Denden I., Stanelle-Bertram S., Beck S., Kouassi N.M., Beythien G., Becker K., Schreiner T. (2022). Microgliosis and neuronal proteinopathy in brain persist beyond viral clearance in SARS-CoV-2 hamster model. EBioMedicine.

[327] Ahmed S., Paramasivam P., Kamath M., Sharma A., Rome S., Murugesan R. (2021). Genetic Exchange of Lung-Derived Exosome to Brain Causing Neuronal Changes on COVID-19 Infection. Mol. Neurobiol..

[328] Rahman A., Tabassum T., Araf Y., Al Nahid A., Ullah M.A., Hosen M.J. (2021). Silent hypoxia in COVID-19: Pathomechanism and possible management strategy. Mol. Biol. Rep..

[329] Butturini E., Boriero D., Carcereri de Prati A., Mariotto S. (2019). STAT1 drives M1 microglia activation and neuroinflammation under hypoxia. Arch. Biochem. Biophys..

[330] Lashgari N.A., Roudsari N.M., Momtaz S., Sathyapalan T., Abdolghaffari A.H., Sahebkar A. (2021). The involvement of JAK/STAT signaling pathway in the treatment of Parkinson’s disease. J. Neuroimmunol..

[331] Musgrove R.E., Helwig M., Bae E.J., Aboutalebi H., Lee S.J., Ulusoy A., Di Monte D.A. (2019). Oxidative stress in vagal neurons promotes parkinsonian pathology and intercellular α-synuclein transfer. J. Clin. Investig..

[332] Wu Z., Zhang X., Huang Z., Ma K. (2022). SARS-CoV-2 Proteins Interact with Alpha Synuclein and Induce Lewy Body-like Pathology In Vitro. Int. J. Mol. Sci..

